# Changes in Oscillatory Dynamics in the Cell Cycle of Early *Xenopus laevis* Embryos

**DOI:** 10.1371/journal.pbio.1001788

**Published:** 2014-02-11

**Authors:** Tony Y.-C. Tsai, Julie A. Theriot, James E. Ferrell

**Affiliations:** 1 Department of Chemical and Systems Biology, Stanford University School of Medicine, Stanford, California, United States of America; 2 Department of Biochemistry, Stanford University School of Medicine, Stanford, California, United States of America; 3 Howard Hughes Medical Institute, Stanford University School of Medicine, Stanford, California, United States of America; Harvard University, United States of America

## Abstract

During the early development of *Xenopus laevis* embryos, the first mitotic cell cycle is long (∼85 min) and the subsequent 11 cycles are short (∼30 min) and clock-like. Here we address the question of how the Cdk1 cell cycle oscillator changes between these two modes of operation. We found that the change can be attributed to an alteration in the balance between Wee1/Myt1 and Cdc25. The change in balance converts a circuit that acts like a positive-plus-negative feedback oscillator, with spikes of Cdk1 activation, to one that acts like a negative-feedback-only oscillator, with a shorter period and smoothly varying Cdk1 activity. Shortening the first cycle, by treating embryos with the Wee1A/Myt1 inhibitor PD0166285, resulted in a dramatic reduction in embryo viability, and restoring the length of the first cycle in inhibitor-treated embryos with low doses of cycloheximide partially rescued viability. Computations with an experimentally parameterized mathematical model show that modest changes in the Wee1/Cdc25 ratio can account for the observed qualitative changes in the cell cycle. The high ratio in the first cycle allows the period to be long and tunable, and decreasing the ratio in the subsequent cycles allows the oscillator to run at a maximal speed. Thus, the embryo rewires its feedback regulation to meet two different developmental requirements during early development.

## Abstract 2

A quantitative study of the *Xenopus laevis* embryonic cell cycle reveals a transition in the first few cycles between two types of oscillator dynamics to meet two different developmental requirements that increase embryo viability.

## Author Summary

The early embryonic cell cycles, which initiate cell division, mark the beginning of the life of an organism. Across different phyla, these cycles have a characteristic temporal pattern, with the first cycle being long and the subsequent cycles shorter, leading to rapid increase in cell numbers. Here we have made use of the *Xenopus laevis* embryos to study the mechanism and significance of this temporal transition. In *X. laevis* embryos, the cell cycles are driven by oscillations in the activity of the cyclin B–Cdk1 complex, which regulates cell cycle progression by protein phosphorylation. We quantified the oscillatory dynamics of key regulators in the first few embryonic cell cycles, and developed an experimentally parameterized mathematical model of the oscillations. We found that a change in the balance between the Cdk1-activating phosphatase Cdc25 and the Cdk1-inhibiting kinases Wee1 and Myt1 is critical for this transition. Tuning this balance converts the cyclin B–Cdk1 oscillator from generating spiky oscillations with delayed activation, to smooth-varying oscillations with a shorter period. Moreover, we found that it is crucial for the first embryonic cell cycle to be sufficiently long, as shortening it with drugs dramatically decreases embryo viability. Our work shows how *X. laevis* embryos modulate their cell cycle oscillator dynamics to meet two developmental requirements: a sufficiently long first cell cycle and rapid progression of the subsequent cycles.

## Introduction

The early embryonic cell cycles mark the beginning of the life of an organism. Across different phyla, including worms [1], flies [2], sea urchins [3], zebrafish [4], and frogs [5], these cycles have a characteristic temporal pattern, with the first cycle being longer and the subsequent cycles shorter. The short cycles result in the rapid accumulation of cells with little or no growth of the embryo.

The *Xenopus laevis* embryo has been a fruitful model system for studies of the regulation of these early embryonic cell cycles. Upon fertilization, the *Xenopus* egg completes meiosis and then carries out a special first mitotic cell cycle. During this cycle the male pronucleus migrates inward from the sperm entry point, the female pronucleus migrates downward from the animal pole, and the two pronuclei congress and proceed through mitosis together. In addition, the cytoplasmic cortex rotates on the side opposite from the sperm entry point to set up the future dorsoventral axis [5]. The first mitotic cleavage then takes place ∼85 min after fertilization. Subsequent divisions occur every ∼30 min in a remarkably precise fashion, with the individual cells within an embryo staying nearly synchronized and the variability in period from embryo to embryo being ∼5% (Table S1). After the 12th division, the embryo proceeds through the midblastula transition, and the rapid embryonic cell cycle is converted into a slower, somatic cell cycle.

**Figure 1:**
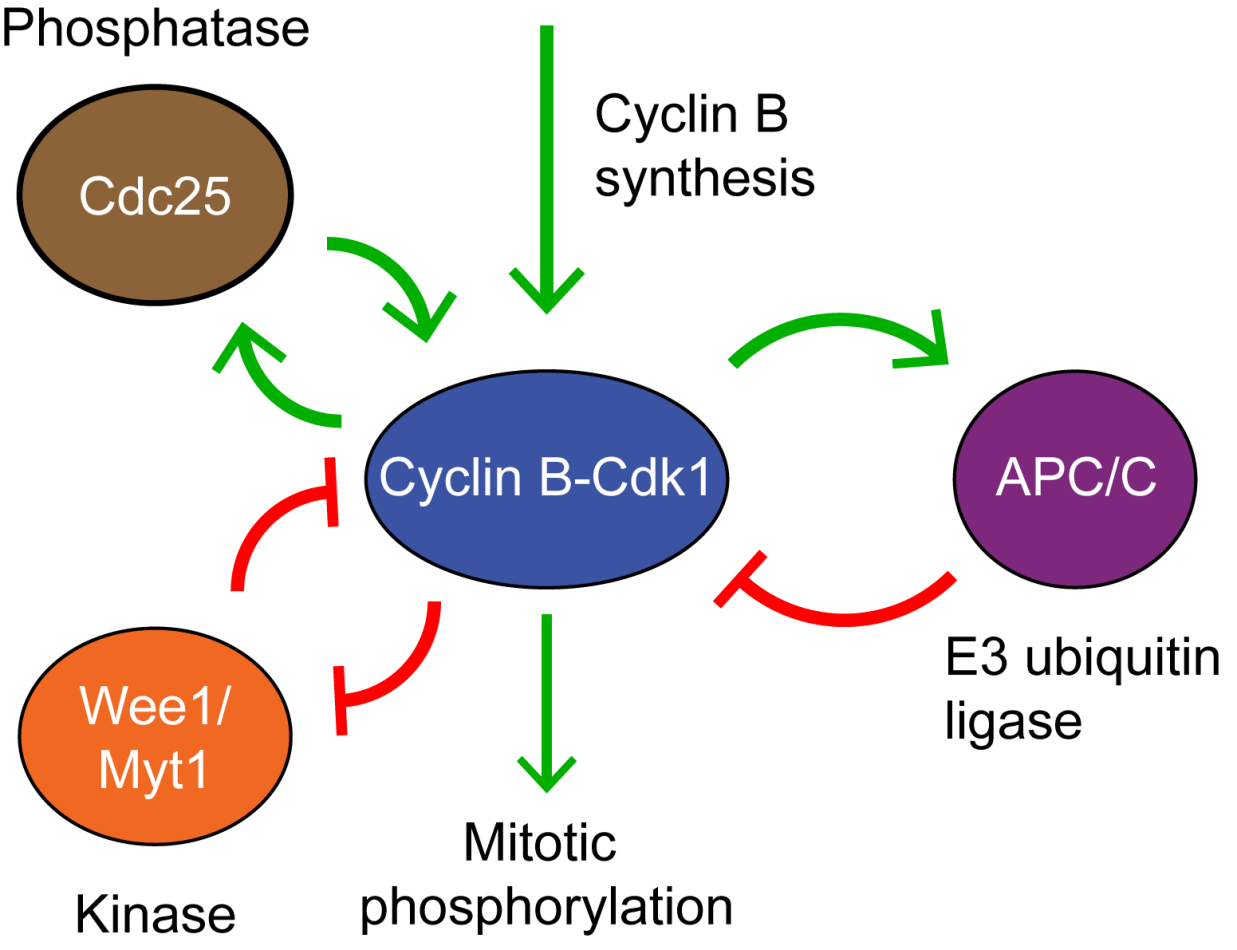
The embryonic cell cycle oscillator consists of interlinked positive-and-negative feedback loops. Cyclin B–Cdk1 inhibits its inhibitory kinases Wee1 and Myt1, forming a double negative feedback loop, which in many respects is equivalent to a positive feedback loop. Cyclin B–Cdk1 activates its activating phosphatase Cdc25, forming a positive feedback loop. Active cyclin B–Cdk1 also activates the E3 ubiquitin ligase APC/C^Cdc20^, which targets cyclin B for degradation. The Cdk1–APC/C^Cdc20^ circuit is therefore a negative feedback loop.

The *Xenopus* embryonic cell cycle is autonomous in character. Cell cycle oscillations persist in the absence of transcriptional activity, DNA replication, and normal microtubule function [6],[7]. The biochemical regulatory circuit that generates these oscillations is centered on the cyclin B-cyclin–dependent kinase 1 (Cdk1) complex, which is the master regulator of mitosis (Figure 1). Cyclin B–Cdk1 is active only when Cdk1 is in the correct phosphorylation state, with Thr 161 phosphorylated and Thr 14 and Tyr 15 dephosphorylated [8]. The kinases Wee1 and Myt1 phosphorylate Thr 14 and Tyr 15 and thereby inactivate Cdk1 [9]–[11]. Both Wee1 and Myt1 are inactivated by Cdk1, forming a double-negative feedback loop [12]–[14], which is similar in many respects to a positive feedback loop. Two phosphatases, Cdc25A and Cdc25C, dephosphorylate Tyr 15 and activate Cdk1 [15]–[18]. In addition, Cdc25C is activated by Cdk1, forming a positive feedback loop [19],[20]. The positive and double-negative feedback loops constitute a bistable trigger [21],[22], and this trigger has been shown through both computational studies [23],[24] and experimental studies [24] to be important for the robustness of the oscillator. Once the bistable switch has been flipped to the Cdk1-on state, Cdk1 activates the anaphase-promoting complex or cyclosome (APC/C), which in *Xenopus* embryos utilizes only the Cdc20 co-activator protein [25]. APC/C^Cdc20^ targets cyclin for degradation by the proteosome. The Cdk1–APC/C^Cdc20^ system constitutes a negative feedback loop, and it is essential for cell cycle oscillations [26].

Although much work has been done on cell cycle regulation in *Xenopus* embryos and extracts, we still lack a quantitative understanding of an important issue. If the periods of cycles 2 to 12 are so precise, what allows the oscillator to be slowed down 2–3-fold in the first cycle? Here we have made use of intact, individual *Xenopus laevis* embryos to quantitatively assess the regulation of the Cdk1 system in fine temporal detail, with the hope of inferring the design principles of the embryonic cell cycle oscillator *in vivo*, and of understanding how the oscillator is remodeled during the transition from the first slow cycle to the rapid subsequent cycles.

## Results

### Reconstructing the Oscillatory Dynamics of Key Cell Cycle Regulators

**Figure 2:**
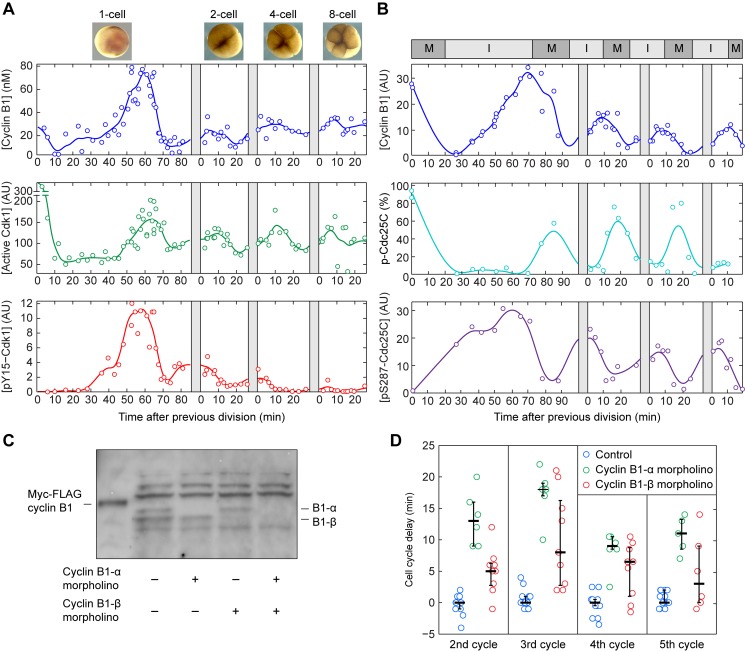
Stronger Tyr 15 phosphorylation in the first cycle results in a longer interphase. (A) Time courses of levels of cyclin B1, Cdk1 activity, and Cdk1 Y15 phosphorylation. The cyclin B1 and pY15–Cdk1 concentrations were measured by quantitative Western blotting, and the Cdk1 activity was measured by histone H1 kinase assay. The original blots are shown in Figure S1D,E. Each point represents a single embryo. For cycles 2–4, relative timing of individual embryos was corrected to the most recent observed cell division, as indicated by the gray bars (see Materials and Methods). (B) Time courses of levels of cyclin B1, hyperphosphorylated Cdc25C, and pSer287–Cdc25C. M-phase and interphase durations are inferred from dynamics of hyperphosphorylated Cdc25C and pSer287–Cdc25C. (C) Evidence for two expressed cyclin B1 genes. Cyclin B1 antibodies [69] recognized two closely spaced cyclin B1 bands, which could be individually knocked down using two different morpholino oligonucleotides. (D) Knocking down cyclin B1-α or cyclin B1-β lengthens the periods of cycles 2–5.

Classic work from Hartley and coworkers established the basic dynamics of cyclin accumulation and Cdk1 activation in early *Xenopus* embryos [27]. Here we set out to extend this work by obtaining quantitative data at higher temporal resolution. We fertilized batches of several hundred *Xenopus* eggs *in vitro*, achieving fertilization efficiencies of >90% and synchronization to within ∼5 min. Individual fertilized eggs were then collected at various times while simultaneously imaging the embryonic divisions. Lysates were prepared from individual embryos; *Xenopus* embryos are large enough (∼1 µL, ∼25 µg protein) that the lysate from a single embryo is sufficient for multiple quantitative Western blots and H1 kinase assays. We optimized the Western blotting and kinase assay protocols to ensure that measurements fell within the linear ranges of the assays (Figure S1). The collection times for individual embryos were expressed relative to the start of the *in vitro* fertilization for the first cell cycle, and to the time of the preceding cleavage for the subsequent cycles (Figure 2A). This *in silico* synchronization limited the time measurement error to <3 min for the second and third cycles.

### Inhibitory Cdk1 Y15 Phosphorylation Occurs Mainly in the First Cycle

**Figure 3:**
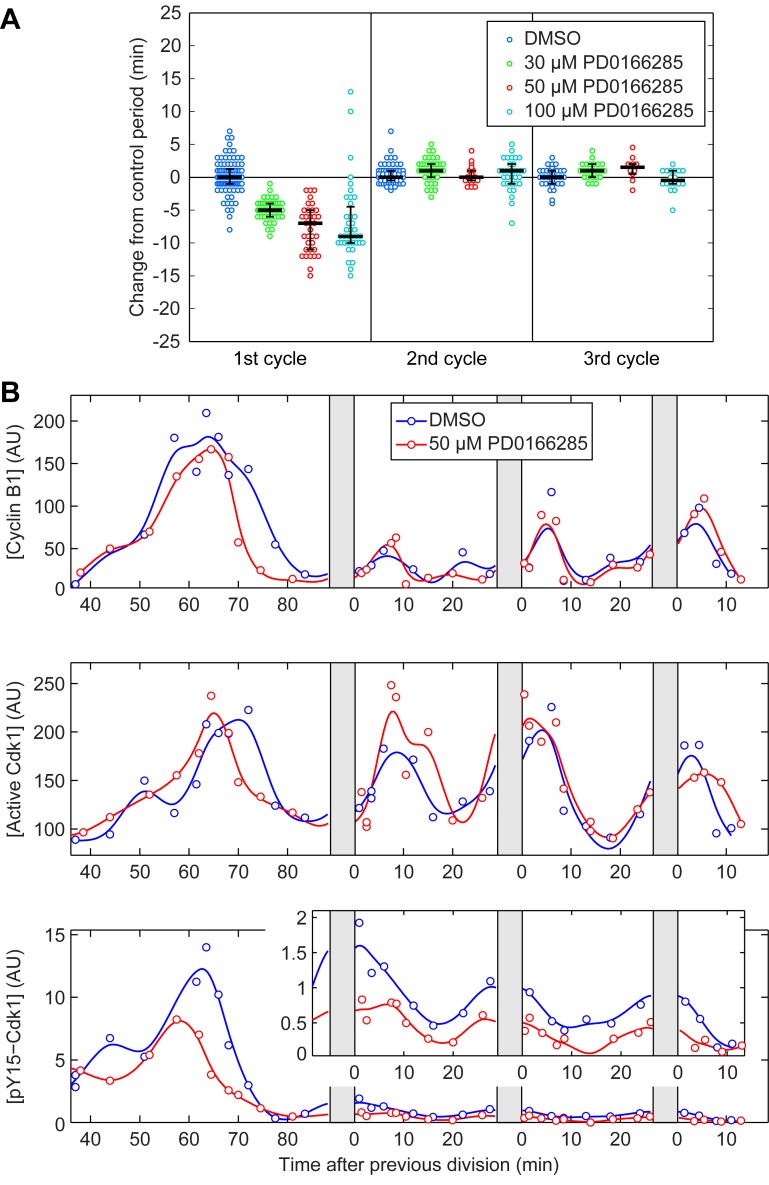
Inhibiting Cdk1 Y15 phosphorylation affects the duration of only the first cycle. (A) The period of the first three cycles from individual embryos treated with PD0166285. All periods are subtracted by the median value of the control of the same cycle to emphasize the differences. The three black lines correspond to 25, 50, and 75 percentile of the population. (B) Time courses of levels of cyclin B1, Cdk1 activity, and pY15–Cdk1 in control embryos and embryos treated with 50 µM PD0166285. (Inset) Higher magnification of pY15–Cdk1 traces from 2nd to 4th period.

Following fertilization there is a period of ∼30 min when cyclin B1 levels decrease and the first meiotic division is completed (Figure 2A,B). At this point the first mitotic cycle begins and cyclin B1 levels rise. During this rising phase, pY15–Cdk1 accumulates, indicating that some fraction of the cyclin–Cdk1 complexes are held inactive by Wee1A and Myt1. Assuming that cyclin B1 degradation is negligible in interphase, the cyclin B1 synthesis rate was estimated to be approximately 1.5 nM/min in all cycles (Figure 2A,B and Figure 3). By 60 min the cyclin B1 levels begin to fall, and ∼10 min before cleavage cyclin levels begin to increase again as the embryos exit their first mitosis.

The peak concentration of cyclin B1 was about 2- to 3-fold higher in the first cycle compared to the subsequent cycles, and the peak level of pY15–Cdk1 was ∼6-fold higher in the first cycle (Figure 2A). In contrast, the peak Cdk1 activity was similar throughout the first five cycles (Figure 2A and Figure 3B). This suggests that the majority of the cyclin B1–Cdk1 complexes are kept inactive by Y15 phosphorylation during the first cycle, and smaller proportions are kept inactive in the subsequent cycles.

To further investigate the length of interphase and M-phase in the first cycle, we assessed the hyperphosphorylation and Ser 287 phosphorylation of Cdc25C. Cdc25C hyperphosphorylation is high during M-phase [28], and Ser 287 phosphorylation is high during interphase [29],[30]. These markers confirmed that interphase was ∼50 min long in the first cycle versus ∼15 min long in the subsequent cycles, whereas M-phase was similar (∼15 min) in duration in all cycles (Figure 2B).

Note that after the first cycle, the oscillations of cyclin B1 levels were fairly modest in amplitude (Figures 2B and 3B). This is reminiscent of the situation in *Drosophila melanogaster* embryos, where the overall abundance of cyclin B is nearly constant during cycles 2–7 [31], and it raises the question of whether these modest oscillations are important for cycling. To address this question we designed morpholino oligonucleotides specific for either of the two *Xenopus laevis* cyclin B1 mRNAs, and knocked down either cyclin B1-α or B1-β (Figure 2C). As shown in Figure 2D, knocking down cyclin B1-α lengthened the cell cycle period by 12.5 ± 1 min (mean ± standard error of the mean, *n*  =  23) and knocking down cyclin B1-β lengthened the cell cycle period by 6.3 ± 1 min (*n*  =  33). These findings support the hypothesis that cyclin B1 accumulation is limiting for cycling in *Xenopus* embryos. Similar conclusions have been drawn based on the ability of cyclin B2 mRNA injection to shorten the cell cycle period [27].

Cdk1 T161 phosphorylation, which is catalyzed by the Cdk-activating kinase (CAK, cyclin H/Cdk7), is required for cyclin B1–Cdk1 complex stability and activation [32],[33]. We therefore asked if this modification varied through the early cell cycles. As shown in Figure S2, Cdk1 T161 phosphorylation correlated well with the measured level of cyclin B1, regardless of whether cyclin B1 levels were rising or falling. Likewise, data from the second and third cycles fell on the same correlation line as data from the first cycle (Figure S2). Thus, the data are consistent with a model where CAK has a constitutive high activity, irrespective of cell cycle phase or number. These findings agree well with earlier studies showing constitutive CAK activity in different types of *Xenopus* extracts [34] and in human cell lines [35].

### Inhibiting Cdk1 Y15 Phosphorylation Accelerates Only the First Cycle

Although the level of pY15–Cdk1 was greatly reduced after the first cycle, it was still detectable (Figure 2A) [17], and this residual level could be functionally significant. To determine the significance of Cdk1 Y15 phosphorylation in the slow first cycle and the subsequent rapid cycles, we treated embryos with PD0166285, a small molecule inhibitor of both Myt1 and Wee1 previously used on mammalian cell lines and *Xenopus* S3 cells [36],[37]. Due to the large size of *Xenopus* embryos, even small molecules permeate slowly and the concentration of the inhibitor in the embryo might be lower than the concentration in the buffer. Therefore, to inhibit Myt1 and Wee1, eggs were pretreated with PD0166285 for 2 to 2.5 h prior to fertilization, and kept in the same concentration of PD0166285 after fertilization and removal of the jelly coat. We observed a dose-dependent reduction in the period of the first cycle by up to 10 min (Figure 3A). Treatment with 50 µM PD0166285 reduced the level of pY15–Cdk1 by half in the first cycle (Figure 3B). Since the inhibitor did not affect the cyclin B1 synthesis rate, the shortening of the first cycle period can be attributed to the decreased inhibitory phosphorylation on Cdk1 (Figure 3B). In contrast, the period in the subsequent cycles remains unchanged after PD0166285 treatment (Figure 3A), although the pY15–Cdk1 levels still decreased by half (Figure 3B, inset). This observation indicates that the inhibitory phosphorylation seen after cycle 1 (Figure 2A) is too low to have a measurable effect on the cell cycle period.

### Multiple Mechanisms Reduce the Effective Wee1-to-Cdc25 Ratio After the First Cycle

**Figure 4:**
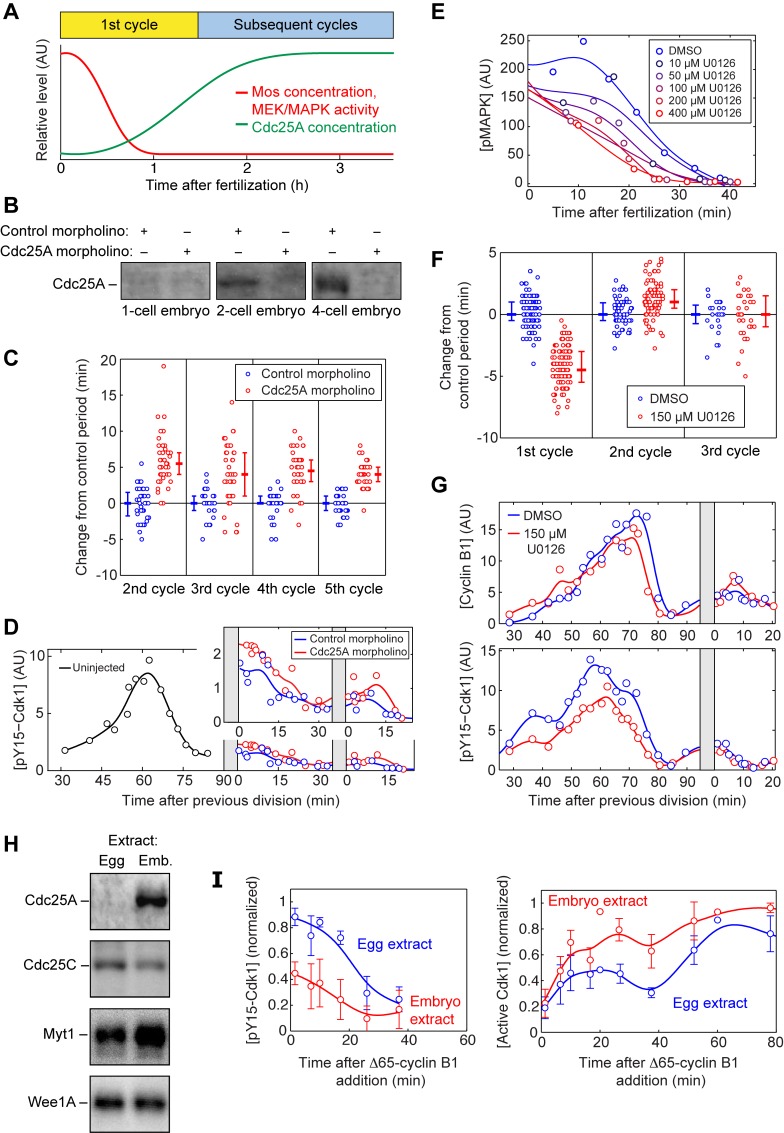
Multiple mechanisms decrease the Wee1/Cdc25 ratio during the transition into the second cycle. (A) Schematic depiction of the decrease of Mos/MEK/MAPK activity, and the increase of Cdc25A concentration, during the first cycle. (B) Cdc25A is absent from one-cell embryos but is present in 2- and 4-cell embryos. The accumulation of Cdc25A is blocked by injection of a Cdc25A morpholino oligonucleotide. The control morpholino is designed with a scrambled sequence of the Cdc25A morpholino. (C) Ablating Cdc25A synthesis causes a small increase in the length of the second through fifth cycles. Fertilized eggs were injected with a Cdc25A morpholino or a scrambled control morpholino. Error bars depict median and 25th and 75th percentiles. (D) Ablating Cdc25A synthesis causes a small increase in pY15 Cdk1 levels in the second and third cycles. The blue points are pY15–Cdk1 levels for embryos injected with a scrambled control morpholino, whereas the red points denote embryos injected with a Cdc25A morpholino. The black points are from uninjected embryos taken during the first cycle. (Inset) Higher magnification of pY15–Cdk1 traces for the 2nd and 3rd periods. (E) The MEK inhibitor U0126 accelerates the postfertilization inactivation of p42-MAPK in a dose-dependent fashion. (F) The period of the first three cycles from individual embryos treated with DMSO or U0126 (150 µM). Error bars depict median and 25th and 75th percentiles. (G) The pY15–Cdk1 level in the first and second cycle. The blue trace denotes the DMSO-treated embryos, and the red trace denotes the U0126-treated embryos. (H) Western blots of Cdc25A, Cdc25C, Myt1, and Wee1A from egg and embryo extracts. (I) Time courses of pY15–Cdk1 and Cdk1 activity after addition of 20 nM Δ65–cyclin B1 to egg and embryo extracts. Data are taken from four experiments. Error bars are standard errors of the mean.

We next set out to examine the molecular mechanisms contributing to the transition from a long first cycle to a short second cycle. An increase in Cdc25 concentration could account for this transition. Indeed, although Cdc25C is present at constant levels throughout the early embryonic cell cycles, Cdc25A is undetectable in unfertilized eggs and rises to half-maximal levels by ∼75 min (Figure 4A) [17]. To test the significance of this newly synthesized Cdc25A, we blocked Cdc25A synthesis by injection of an antisense morpholino oligonucleotide. The morpholino blocked Cdc25A accumulation (Figure 4B), lengthened the period of the cell cycle by about 5 min (Figure 4C), and slightly increased the Y15-phosphorylation of Cdk1 (Figure 4D). However, the pY15–Cdk1 levels were still significantly lower and the cell cycle periods still significantly shorter than those seen in the first cycle. This indicates that Cdc25A is not required for cycling. Interestingly, in mouse knockout experiments, Cdc25A is essential in embryogenesis, whereas Cdc25B and Cdc25C are not [38]. However, in *Xenopus* embryos Cdc25A appears to be inessential and Cdc25A accumulation only partially accounts for the shortening of the cell cycle after the first division; some additional mechanisms must also play a role.

Another possible mechanism would be a decrease in the amount or activity of Wee1 and Myt1. Previous work on *Xenopus* egg extracts suggested that Wee1 may be more active during the first cycle than in subsequent cycles because of positive regulation of Wee1 by the Mos/MEK/p42 MAPK cascade, which is substantially more active in the first cycle than in subsequent cycles [39]–[41]. However, the cascade also has been reported to negatively regulate Myt1 [14], and so the net quantitative effect on the total Wee1-plus-Myt1 activity is uncertain. To test whether the MAPK cascade helps establish a long first cycle in intact embryos, we carried out experiments with the MEK inhibitor U0126. Consistent with previous extract studies [40], we found that it was difficult to completely inactivate MEK in unfertilized eggs; even with a maximal dosage of U0126 (400 µM), we only achieved a partial reduction in MAPK activity and accelerated the postfertilization inactivation of p42 MAPK by 10 min (Figure 4E). Nevertheless, partial inactivation of MAPK with 150 µM U0126 shortened the period of the first cycle by 5 min (Figure 4F), and reduced the peak level of pY15–Cdk1 by ∼30% (Figure 4G). The subsequent cell cycle periods stayed unchanged in the presence of U0126. This suggests that the Mos/MEK/p42 MAPK cascade has a net positive effect on Cdk1 Y15 phosphorylation, and that this effect is lost after Mos is degraded during the first cell cycle.

Thus, it appears that MEK activity (Figure 4F) and Wee1 activity (Figure 3) contribute to slowing the first cycle, and the synthesis of Cdc25A (Figure 4A–C) contributes to the rapidity of the subsequent cycles. However, we have not been able to make the first cycle as rapid as subsequent cycles with pharmacological inhibitors (U0126, PD0166285, or combinations). This could simply be due to incomplete inhibition, although it remains possible that unidentified factors contribute to the difference in length of the first versus subsequent cycles.

Finally, we set out to determine whether the differences between the first cycle and the subsequent cycles in an intact embryo could be recapitulated in extracts *in vitro*. We prepared cycloheximide-treated interphase extracts from parthenogenetically activated eggs (hereafter referred to as egg extracts), and from embryos passing the two-cell stage (embryo extracts), in a manner similar to that described by Kubiak and coworkers [42]. The two types of extract possessed similar concentrations of Wee1A and Cdc25C, whereas Myt1 was somewhat higher in embryo extracts, possibly because more Myt1 is lost during the centrifugation of interphase egg extracts than embryo extracts (see details in Materials and Methods). In addition, Cdc25A was present only in embryo extracts, as expected (Figure 4H). We added sufficient nondegradable Δ65–cyclin B1 to drive the extracts into mitosis, and compared the extracts’ responses.

As shown in Figure 4I, the egg extracts responded with higher levels of pY15–Cdk1 and lower levels of Cdk1 activity than did the embryo extracts. These findings indicate that the effective Wee1-to-Cdc25 ratio is higher in the egg extracts than in the embryo extracts. We also detected strong p42 MAPK phosphorylation in mitotic egg extracts, but not in embryo extracts or intact embryos (Figure S3 and unpublished data). The p42 MAPK phosphorylation seen in egg extracts was blocked by the MEK inhibitor U0126 (Figure S3). This finding underscores the observation that residual Mos is present during the first cell cycle and absent from later cycles [43],[44]. Taken together, these observations indicate that *Xenopus* egg extracts as usually prepared, with their relatively high levels of Mos and low levels of Cdc25A, may more accurately reflect the biochemical environment of the first cycle than the subsequent cycles. Conversely, embryo extracts, with lower Mos levels and higher Cdc25A levels, may be a more appropriate *in vitro* system for studies of the subsequent cell cycles.

### Modeling the Embryonic Cell Cycle

Mathematical modeling can provide insight into the design principles of the embryonic cell cycle [23],[45]. The first ordinary differential equation (ODE) models of the *Xenopus* embryonic cell cycle were proposed more than 20 years ago [45]–[47]. Although the basic framework established by Tyson and Novak still holds up remarkably well [45], we have now learned a substantial amount about the quantitative behavior of the components of the cell cycle oscillator that was not known then [48]–[50]. We therefore set out to see whether we could model the embryonic cell cycle and account for the transition from a slow first cycle to rapid subsequent cycles, given what we now know about the process.

As described in Text S1, we formulated a 4-ODE model that strips the Cdk1–APC/C circuit down to its essential core. The model accounts for the synthesis and degradation of mitotic cyclins, the regulation of Cdk1–cyclin B1 complexes by Wee1 and Cdc25, and the two-step activation of APC/C^Cdc20^ (with two steps included to produce a realistic time lag between Cdk1–cyclin B1 activation and APC/C^Cdc20^ activation) [50]. Two key features contributed to the generation of oscillations in the model: the presence of a bistable trigger (from the Cdk1–Wee1–Cdc25 positive and double-negative feedback loops) and the presence of ultrasensitivity in the Cdk1–APC/C^Cdc20^ negative feedback loop. Both of these features have been validated experimentally for the first cycle through experiments in egg extracts [22],[50]. However, in light of the fact that the bistable trigger is altered after the first cycle, we set out to determine experimentally whether the other key element, the ultrasensitivity in the negative feedback loop, persists.

**Figure 5:**
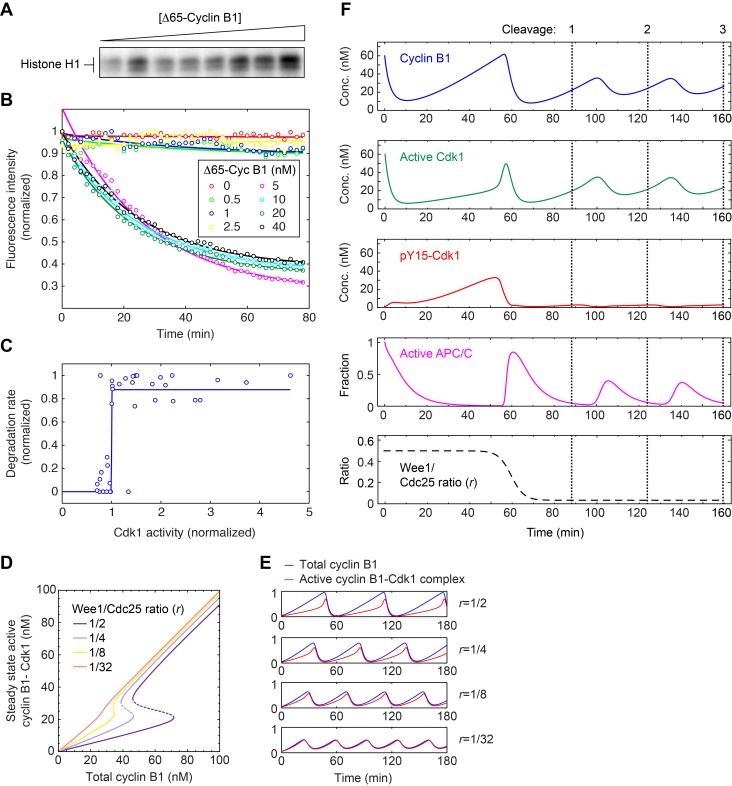
Constructing an ODE model of the embryonic cell cycle. (A–C) Ultrasensitive negative feedback in embryo extracts. Interphase embryo extracts were treated with PD0166285 and various concentrations of ▵65–cyclin B1, yielding a graded range of Cdk1 activities as assessed by histone H1 phosphorylation (A) and an all-or-none response in the degradation of securin-CFP, an APC/C^Cdc20^ substrate (B). The inferred stimulus/response curve for securin degradation as a function of Cdk1 activity (C) was highly ultrasensitive, with a best-fit Hill exponent of 464 and 90% confidence interval of (55,539). Data are taken from five experiments. (D) Calibrating the positive feedback strength by varying the Wee1 versus Cdc25 activity ratio (*r*). Several assumed values of *r* are shown. A value of 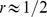 corresponds well to the bistability observed in experiments on *Xenopus* egg extracts and a physiologically strength of positive feedback [22]. After the first cycle, *r* decreases to approximately 1/32; see Figure S3. (E) Oscillations at various assumed positive feedback strengths. (F) Modeling the transition from the first cycle to the subsequent cycles by adjusting only the positive feedback strength (*r*). Compare to Figure 2A.

We added different concentrations of Δ65–cyclin B1 to interphase embryo extracts in the presence of PD0166285, which allowed us to achieve various graded levels of Cdk1 activity (Figure 5A). After the extracts approached steady state, we added securin-CFP, an APC/C^Cdc20^ substrate, and monitored its rate of degradation. We found that the securin-CFP protein switched from no degradation to maximal degradation over a narrow range of Δ65–cyclin B1 concentrations (Figure 5B,C), indicating a high degree of ultrasensitivity. To quantify how ultrasensitive the response was, we fitted a Hill equation to the pooled and scaled degradation rate data. The best-fit Hill exponent was 464, a huge number, and the 90% confidence interval by bootstrapping was 55 to 539 (Figure 5C). Thus, the observed response was essentially all-or-none in character. This indicates that the *Xenopus* cell cycle oscillator operates with a highly ultrasensitive negative feedback loop in both the first cycle and subsequent cycles. Several mechanisms could account for this highly switch-like behavior [50]–[53].

Interestingly, the ultrasensitivity of the negative feedback measured in embryo extracts was even higher than the ultrasensitivity reported in egg extracts [50]. This suggests that the *Xenopus* embryo may increase the ultrasensitivity of the negative feedback after the first cycle, possibly to compensate for the loss of positive feedback.

### A Modest Reduction in the Wee1-to-Cdc25 Ratio Effectively Eliminates Positive Feedback

With the basic framework of the computational model set, we next examined how changing the balance between Wee1 and Cdc25 activity would affect the behavior of the model. Both of the mechanisms examined in Figure 4, the accumulation of Cdc25A and the inactivation of MAPK, would alter the balance in favor of Cdc25. Operationally we defined the balance by a single parameter, the ratio 
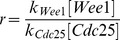
, because it was the balance between Wee1 and Cdc25 rather than the absolute levels of either that mattered most in the modeling. With our parameterization, an *r* value of ∼1/2 produced a bistable curve for the steady state response of Cdk1 to cyclin B1 that was comparable to that found experimentally [22], and this value yielded cycles of Cdk1 activation and inactivation similar to those seen in the first cell cycle in embryos (Figure 2A,B).

We took two approaches to the estimation of the value of *r* after the first cycle, based on the amplitude of pY15–Cdk1 oscillations and on the relationship between cyclin B1 and pY15–Cdk1 during interphase (Figure S4). Both approaches suggested that *r* falls by ∼16-fold, to a value of ∼1/32. This value of *r* made the modeled response of Cdk1 to cyclin B1 nearly linear (Figure 5D) and made the oscillations more sinusoidal (Figure 5E), like those typical of negative-feedback-only oscillator circuits. For the sake of brevity we can refer to the cell cycle oscillator model as being effectively a negative-feedback-only circuit when *r*  =  1/32, although it should be borne in mind that the positive and double-negative feedback loops have not actually been eliminated, but only changed with respect to their balance.

To reproduce the switch from the slow first cycle to the rapid subsequent cycles, we assumed the value of *r* was initially 1/2 and then fell during the first cycle to 1/32. This succeeded in reproducing the transition from spiky to smooth oscillations in Cdk1 activity (Figure 5E and F). The other key features of the embryonic cell cycle seen experimentally were captured as well: the higher level of cyclin B1 and Cdk1 Y15 phosphorylation in the first cycle, the extended interphase of the first cycle, and the relative speeds of the first versus subsequent cycles (Figures 2A,B and 5F). Thus, the transition from the slow first cycle to the rapid second cycle can be accounted for on the basis of a relatively modest change in the balance between Cdc25 and Wee1.

### Dissecting the Modeled Slow and Fast Cycles

The ODE model makes it possible to study and compare the systems-level properties of the oscillator when it operates in the long period, positive-plus-negative feedback regime, and the short period, negative-feedback-only regime. First we examined the robustness of oscillations in the face of changes in the models’ parameter values. This may be relevant to the challenges the oscillator circuit must deal with *in vivo* in the face of an inconstant environment. For example, embryos must be able to develop over a range of ambient temperatures. We have found that the early embryonic cell cycle proceeds normally at temperatures ranging from 12–28°, even though cyclin synthesis and the period of the cell cycle vary by ∼6-fold (120 min to 20 min). Unless every enzyme in the cell cycle oscillator circuit has exactly the same temperature dependence, it is likely that the circuit is tolerating substantial changes in the relative activities of its components.

**Figure 6:**
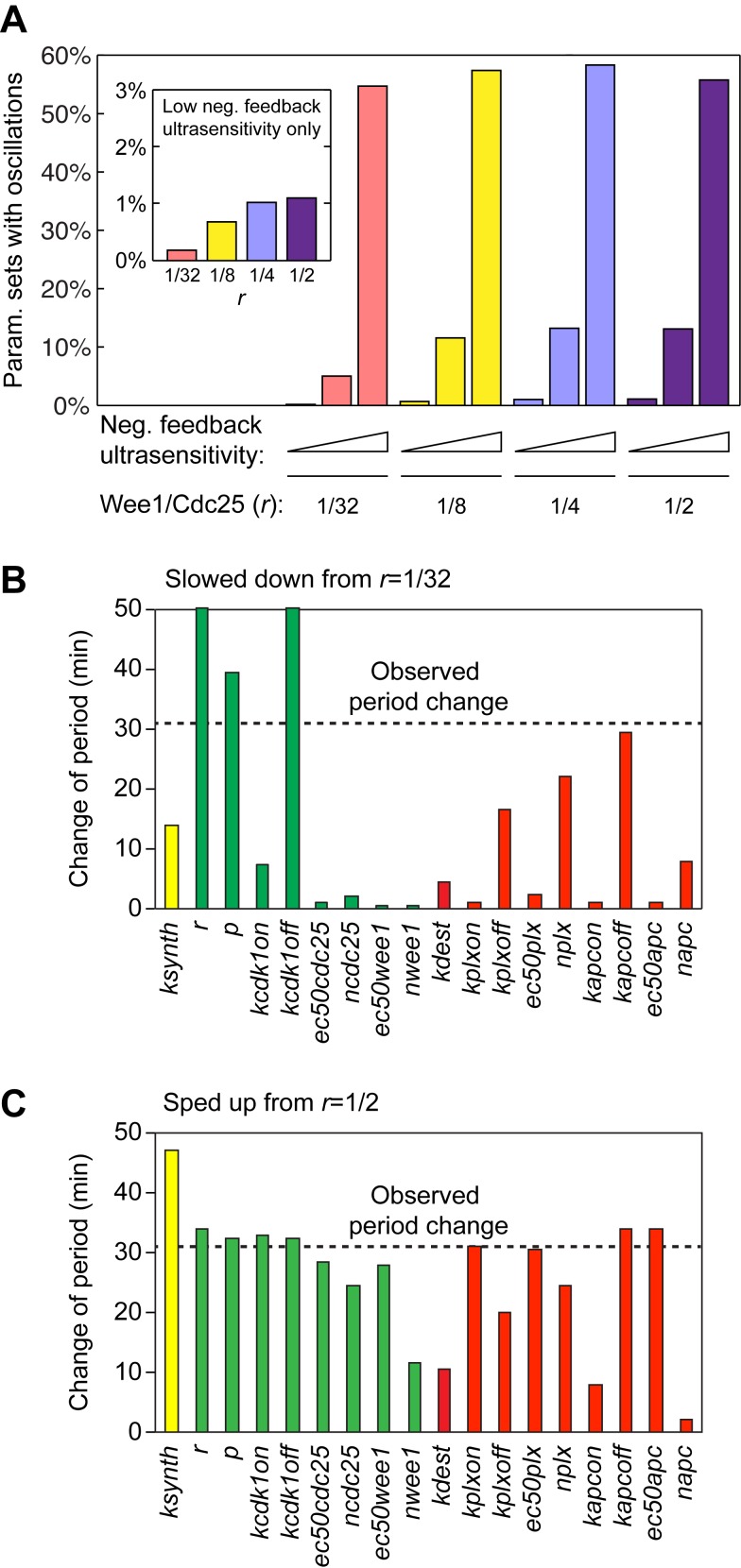
Modeled robustness and tunability from negative-feedback-only versus positive-plus-negative feedback. (A) Robustness score of the oscillator assuming various degrees of ultrasensitivity in the negative feedback loop (*n*  =  4, 9, or 36; see Text S2), and various values of *r*. (B, C) Tunability. Each of the model’s parameters was varied up and down by 32-fold, starting with a value of *r* that made the model run like a negative-feedback-only oscillator (*r*  =  1/32, panel B) or a positive-plus-negative feedback oscillator (*r*  =  1/2, panel C). The bars show the maximum increases (B) and decreases (C) in period that resulted. The green bars correspond to parameters related to the positive feedback, the red bars to negative feedback, and the yellow bars represent cyclin synthesis.

Previous modeling studies and experimental studies have shown that a bistable trigger can contribute robustness to the generation of oscillations, and it has been argued that this might be one reason why positive-plus-negative feedback designs are so common in biological oscillators [23],[24],[54],[55]. However, the cell cycle appears to proceed reliably and with a very regular period in *Xenopus* embryos during cycles 2–12, where the bistable trigger is essentially inoperative (Figures 2–4). We therefore set out to determine whether the highly ultrasensitive activation of APC/C^Cdc20^, which we now know to be present in the embryonic cell cycle [50], might obviate the need for a bistable trigger. As a measure of robustness, we randomly varied the model’s parameters and scored the percentage of parameter sets that yielded oscillations (Text S2). As shown in the inset to Figure 6A, when relatively low levels of ultrasensitivity were assumed for the negative feedback loop, positive feedback contributed to the robustness of oscillations. However, if we assumed realistically high levels of ultrasensitivity in the negative feedback loop, the oscillations were extremely robust irrespective of whether we assumed a high or low value for *r* (Figure 6A). This finding implies that even though a bistable trigger can promote oscillations, high ultrasensitivity in the negative feedback loop may make a bistable trigger inessential. Thus, both the strong and weak positive feedback versions of the model can generate robust oscillations.

Finally, we asked whether changing the positive feedback strength may be a particularly effective way of converting a long first cycle into a short second cycle. First we asked whether a negative-feedback-only (*r*  =  1/32) oscillator could have been used for both the long first cycle and the short subsequent cycles without strengthening the positive feedback. We tuned each of the model parameters up and down by 32-fold and asked which individual parameters could lengthen the cycle by 31 min, the approximate difference between the first mitotic cycle and the second. We found that three parameters (*r*, *p*, and *kcdk1off*) could achieve this extent of slowing, and all three were related to the strength of the positive feedback (Figure 6B). Thus, only those manipulations that increased positive feedback strength allowed the oscillations to be slowed without being extinguished.

Next we examined whether a positive-plus-negative feedback (*r*  =  1/2) oscillator could have been used for both the long first cycle and the short subsequent cycles without weakening its positive feedback. As shown in Figure 6C, eight different parameters could shorten the cycle by 31 min, including the positive-feedback-related parameters, but including other parameters as well. Thus, adjusting the strength of the positive feedback is one of several effective ways of transitioning from a slow cycle to a rapid cycle. Strong positive feedback allows the first cycle to be adjusted to whatever period is required, and eliminating the positive feedback allows the cell cycle to run as fast as it can.

Additional analysis of the model can be found in Figure S5 and its legend.

### A Slow First Cycle Is Crucial for Embryo Viability

**Figure 7:**
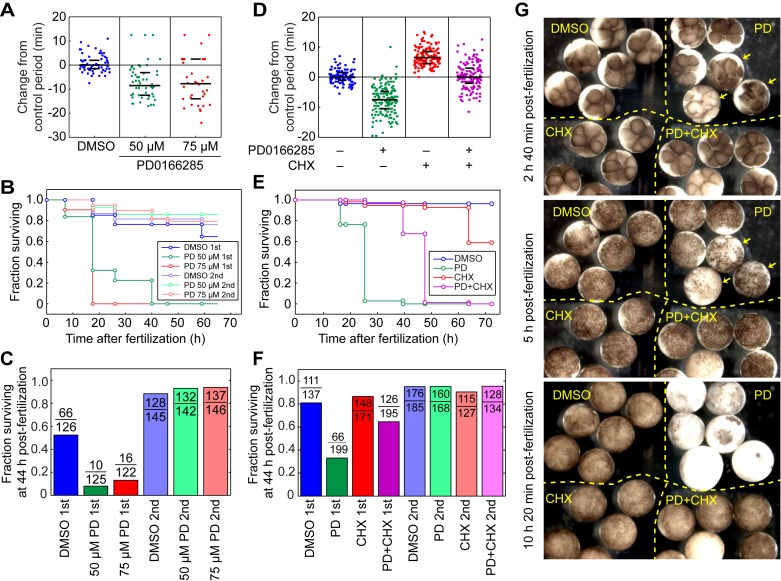
Shortening of the first cycle period significantly reduces embryo viability, and cycloheximide rescues viability. (A–C) Application of PD0166285 during the first cycle causes a loss of viability, whereas later treatment does not. (A) Changes in the length of the first cycle in response to two concentrations of PD0166285. (B) Kaplan–Meier survival curves. (C) Survival at 44 h postfertilization. The data in (A) and (C) are from four experiments, whereas the data in (B) are from one representative experiment. (D–F) Cycloheximide (CHX, 0.25 µg/mL) partially rescues the effects of PD0166285 (30 µM) on viability. (D) Changes in the length of the first cycle in response to PD0166285 ± CHX. (E) Kaplan–Meier survival curves. (F) Survival at 44 h postfertilization. The data in (D) and (F) are from four experiments, whereas the data in (E) are from one representative experiment. (G) Photographs of drug-treated and control embryos at various times after fertilization. The embryos were placed in the same petri dish after the inhibitors were washed out at the completion of the first cycle. The arrows designate three PD-treated embryos that have discoordinated cell divisions as early as a few hours postfertilization; the other PD-treated embryos are grossly normal until the midblastula transition (bottom panel). The incubation temperature was 18° for the experiments in (A–F), and 23° for the experiment in (G).

Finally, we asked whether a slow first cycle is important for proper embryonic development. To this end, we inhibited Wee1 and Myt1 with PD0166285 during the first cycle only and observed the developmental consequences. PD0166285 treatment decreased the period of the first cycle by 10 min (Figure 7A; see also Figure 3A). At the conclusion of the first cycle, the PD0166285 was washed out and the survival of the embryos was recorded through the tadpole stage. Most of the PD0166285-treated embryos completed the early embryonic cell cycles without problem, but viability dropped significantly at the midblastula transition (Figure 7B) and less than 1% of the embryos successfully developed into tadpoles (Figure 7C). In contrast, embryos treated with PD0166285 for the same duration after completion of the first cycle developed normally, indicating that the PD0166285-mediated effect was specific to the first cycle (Figure 7B,C).

To determine whether the reduction of viability was due to the shortening of the period of the first cycle, we treated the embryos with both PD0166285 (30 µM) and a low concentration of cycloheximide (0.25 µg/mL). Cycloheximide inhibited cyclin synthesis and lengthened the cell cycle (Figure 7D, Figure S6); hence, it might reverse the PD0166285-induced loss of viability if the loss is due simply to shortening of the first cycle. We chose a concentration of cycloheximide that was just high enough to restore the first cycle’s period to a normal length (Figure 7D). We then treated embryos with PD0166285 ± cycloheximide, washed out the inhibitors after the first cleavage, and then assessed embryo viability as a function of time. As shown in Figure 7G, some of the embryos treated with PD0166285 underwent discoordinated cell divisions early on. However, regardless of whether the early embryos were grossly normal or abnormal, at the midblastula transition most of the PD0166285-treated embryos underwent embryonic death (Figure 7E–G and Movie S1). The death phenotype was largely rescued by co-treatment with cycloheximide during the first cycle (Figure 7E–G and Movie S1). This finding implies that an essential function of Wee1/Myt1 in the first cycle is to keep the cycle from being too short. These data also suggest that the first cycle period in the wild-type *Xenopus laevis* embryos might already be approaching the shortest permissible limit, because further shortening of this period resulted in massive embryonic death.

Note that although PD0166285 and cycloheximide co-treatment rescued the PD0166285 phenotype as assessed at the midblastula transition, the co-treated embryos still generally failed to elongate at the tailbud stage and died before becoming tadpoles (Figure 7E–G). Thus, in addition to lengthening the first cycle, the Wee1/Myt1 kinases may have other functions during the first cycle that become important in later stages of embryonic development.

## Discussion

By characterizing the temporal dynamics of key cell cycle regulatory proteins in the *Xenopus laevis* embryo and comparing the results with computational models, we have obtained a quantitative understanding of the transition that occurs between the first and subsequent cell cycles. The long first cycle is driven by a regulatory circuit that includes both a bistable, positive feedback trigger and an ultrasensitive negative feedback loop (Figures 1 and 2). In the subsequent cycles, the positive feedback is effectively eliminated, even though Wee1A and Cdc25 are still present and are still regulated (Figures 2 and 3). The elimination of the positive feedback is achieved by a shift in the balance between Wee1 and Cdc25 activities, in part because of the synthesis of Cdc25A after the first cycle and in part because the Mos/MEK/p42 MAPK cascade positively regulates Wee1A only during the first cycle (Figure 4). Positive feedback allows the oscillator to be tuned to a long period in the first cycle (Figure 6), while eliminating the positive feedback allows the subsequent cycles to proceed as quickly as possible (Figure 6). This long first cycle is of critical importance for proper embryo development; speeding the first cycle up by PD0166285 treatment greatly affects subsequent development, and slowing the cycle back down with cycloheximide rescues viability (Figure 7).

These findings raise the question of why a slow first cell cycle is important. During the first cycle, the sperm pronucleus migrates through the egg cytoplasm over distances of hundreds of microns to meet and fuse with the egg pronucleus. This congression is complete by approximately 50 min after fertilization. At around the same time, the gray crescent forms as a result of cortical rotation to set up the future dorsal-ventral axis [5]. We suspect that development is compromised in the PD0166285-treated embryos because of a failure to complete congression, cortical rotation, or some other G2-phase event (like chromatin condensation or centrosome maturation). A long first embryonic cell cycle is commonly observed across multiple species, from *C. elegans* [1], *Drosophila* [2], sea urchin [3], zebrafish [4], to *Xenopus*. Although the period of early embryonic cell cycles in mammals is longer and comparable to somatic cell cycles, the first cell cycle of mouse and human embryos is still longer than the subsequent ones [56],[57]. It remains to be determined whether shortening the first cell cycle in other species has an impact as profound as in *Xenopus laevis*. A comparison of the strategies utilized in different organisms to extend their first embryonic cell cycle may provide insight into the evolution of this ancient machinery.

The Wee1/Myt1/Cdc25 bistable trigger is well-conserved from yeast to humans. In many organisms and cell types, this circuit is critical for normal cell cycle regulation. For example, in *S. pombe*, mutations in Wee1 and Cdc25 cause marked changes in cell size [58],[59]; in *Xenopus* extracts, depleting Wee1 makes Cdk1 oscillations less sustained [24]; and in HeLa cells, Wee1 knockdown results in markedly abnormal rapid cycling [60]. However, in some organisms and cell types, the circuit is dispensable. In *S. cerevisiae*, the effects of Wee1 (Swe1) deletion are more subtle than they are in *S. pombe* [61], and even in *S. pombe*, strains can be engineered that cycle fairly normally in the absence of Wee1 and Cdc25 [62]. *Xenopus* embryos provide a natural example of a system where, depending on context, Wee1 may or may not be essential; it is essential during the first cycle—its inhibition causes death later in embryonic development—and then becomes dispensable in the subsequent cycles.

Oscillators built on interlinked positive and negative feedback loops are widespread in biology. The combination can allow oscillators to have highly tunable periods [23]. Many biological oscillators do operate over a range of periods, and interlinked positive-and-negative feedback loops are indeed central to the regulation of these systems [63]. Examples of biological oscillators that operate solely on ultrasensitive negative feedback are less numerous, although negative-feedback-only models of circadian oscillations, NFκB oscillations, and myxobacterial swarming have all been proposed [64]–[66]. The *Xenopus* early embryonic cell cycle oscillator represents a hybrid of the two categories, with the tunability of the positive-plus-negative feedback design obtained initially and the speed of the negative-feedback-only design achieved subsequently. In this way, the oscillator circuit is developmentally remodeled to satisfy two different performance goals.

## Materials and Methods

### Collecting Single *Xenopus laevis* Embryos and Reconstructing Temporal Dynamics

All animal work was conducted according to relevant national and international guidelines. Animal protocols were approved by the Stanford University Administrative Panel on Laboratory Animal Care. Female *Xenopus laevis* were induced by human chorionic gonadotropin injection and pelvic massage was performed to collect eggs 12 to 20 h after induction. For pretreatment with chemical inhibitors, eggs were directly squeezed into high salt buffer and incubated at 18–22° for 2–2.5 h. *In vitro* fertilization was performed by mixing eggs with smashed testis for 2 min and flooding with 0.1X Marc’s Modified Ringer’s (MMR) buffer with or without inhibitors. Fertilized embryos were treated with 2% cysteine in 0.1X MMR for 3–5 min about 15–20 min after fertilization to remove the jelly coat. After cysteine treatment, embryos were washed with 0.1X MMR three times and then placed in 0.1X MMR buffer with or without inhibitors. The embryonic divisions were imaged with a stereoscope Nikon SMZ 1500 with a Leica DFC425 camera. Frame rate was 1 frame/30 s. Individual embryos were collected with an inoculation loop (Kendall Bioloop) at various time points, labeled and placed in microcentrifuge tubes, and frozen on dry ice.

*In silico* synchronization was performed by assigning each collected embryo with a corrected time according to the time difference between the time of collection and its previous division. After completion of sample collection, individual embryos were lysed with EB buffer, centrifuged in thin microcentrifuge tubes (E&K485050) to separate lipid and yolk from the cytoplasm, and the cytoplasmic portion was collected. Typically, 1/3 to 1/2 of the embryo lysate from a single embryo was sufficient for a Western blot, and 1/10 of the single embryo lysate was sufficient for a histone H1 kinase assay.

### Quantitative Western Blotting and Kinase Assays

Histone H1 kinase assays were carried out as described [67]. ^32^P-labeled histone H1 was detected using a Phosphorimager Storm 840 (GE Healthcare). Care was taken to ensure the linearity of the assay, and to make sure that the exposures of phosphohistone bands on different gels were equivalent (Figure S1). For immunoblotting, samples were separated by PAGE, transferred to a PVDF membrane, incubated with antibodies, visualized by enhanced chemiluminescence (Thermo Scientific) and Gel Doc imaging (BioRad, Hercules CA), and quantified using ImageJ. Again, care was taken to ensure linearity and equal exposure (Figure S1). In the case of cyclin B1, intensities were converted to absolute units (nM) using purified recombinant cyclin B1 as a standard protein. For other proteins, the results were expressed in arbitrary units (AUs).

### Antibodies, Morpholinos, and Inhibitors

The cyclin B1 antibody was a gift from Julian Gannon and Tim Hunt. The pY15–Cdk1 antibody was from Cell Signaling (9111). The Wee1 antibody was from Invitrogen (511700). The Cdc25C antibody was raised by Nikki Trunnell [49]. The pSer287–Cdc25C antibody was a gift from Joan Ruderman [29]. The Cdc25A antibody was a gift from James Maller, and the Myt1 antibody was a gift from William Dunphy.

The Cdc25A morpholino (Gene-Tools) was designed to target the Cdc25A NM_001096204 and NM_001088487, with the 5′ to 3′ sequence being GAGCAGAGCGACACCTCTCCATCCT and GAGCAGAACGAAACCTCTCCATCTT. The control morpholino sequence was GACCACAACCAAACCTGTGCATCTT. Cyclin B1-α corresponds to the cDNA sequence NM_001086727.1, and the morpholino was ACATTTTCCCAAAACCGACAACTGG. Cyclin B1-β corresponds to the cDNA sequence NM_001087797.1, and the morpholino was ACATTTTCTCAAGCGCAAACCTGCA.

The MEK inhibitor U0126 was obtained from Cell Signaling (9903). The Wee1/Myt1 inhibitor PD0166285 was obtained from the compound transfer program of Pfizer.

### *Xenopus* Egg and Embryo Extracts

*Xenopus* egg and embryo extracts were prepared based on modifications of previous protocols [67],[68]. For *Xenopus* egg extracts, freshly squeezed eggs were treated with 2% cysteine for 5 min to remove the jelly coat. Dejellied eggs were activated parthenogenetically by calcium ionophore A23187 (1 µg/ml) (Sigma) for 2 min, and incubated with 0.1X MMR buffer [67] for 20–30 min before starting the preparation of the extract. For *Xenopus* embryo extracts, eggs were squeezed into a 6-cm dish containing smashed testis. Fertilized eggs were flooded with 0.1X MMR 2 min after mixing with the testis sample. The fertilized eggs were treated with 2% cysteine to remove the jelly coat 20–30 min after fertilization. Eggs failing to form cleavage furrows 90 min after fertilization were considered unfertilized and discarded. We typically waited 2–2.5 h after fertilization to start preparation of the embryo extracts.

To prepare the interphase egg and embryo extracts, we adapted the standard extract procedures to smaller volumes. Eggs or embryos were washed 3 times in egg lysis buffer (50 mM KCl, 2.5 mM MgCl_2_, 250 mM sucrose, 10 mM HEPES, pH 7.7). After the last wash, we supplemented the buffer with a cocktail of inhibitors [protease inhibitors leupeptin, chymostatin, pepstatin, each at a final concentration 5 µg/ml, actin inhibitor cytochalasin B (10 µg/ml), and for interphase extracts, we added the translation inhibitor cycloheximide (50 µg/ml)]. Eggs or embryos were loaded into thin microcentrifuge tubes (E&K485050) containing 30 µl of mineral oil and were packed by low speed centrifugation. Fertilized embryos are more rigid than unfertilized eggs, and consequently a harder spin was required for the embryo extracts (5 min at speed setting 5 for embryos and 3 min at speed setting 3 for eggs, in a Damon IEC 6-Place Clinical Centrifuge). Excess buffer and mineral oil was removed, and the eggs/embryos were sheared by centrifugation [15 min at 15,000 g in a right angle rotor (Beckman Microfuge E)]. After the spin, we used a razor blade to cut away the lipid layer and aspirated the cytoplasmic portion of the extract carefully to prevent mixing with the yolk layer. Typically, we obtained ∼50 µl of cytosolic extracts from each tube, and we combined extracts from 4–6 tubes for each experiment.

Note that the centrifugation time and speed in the final step of extract preparation affected the Myt1 concentration in the extracts. Myt1 is a membrane-associated protein, and longer centrifugation caused more of the Myt1 to be lost to the pigment granule/yolk fraction. We typically obtained less Myt1 in cytoplasmic extracts when embryos were in interphase than in M-phase, possibly due to the fragmentation of the endoplasmic reticulum in M-phase. The parthenogenetically activated eggs were typically in interphase synchronously at the time of extract preparation, while the embryos were in a mixed state. Therefore, with identical centrifugation setting, we typically saw more Myt1 retained in the embryo extracts than in the egg extracts.

### Real-Time Fluorescence Assay for APC/C^Cdc20^ Substrate Degradation in *Xenopus* Embryo Extracts

The detailed methods were described previously [50]. Briefly, we constructed cDNAs for securin-CFP, an APC/C substrate, and expressed it *in vitro* in a wheat germ system (TNT SP6 High Yield Protein Expression, Promega). A small amount (0.5–1 µl out of the 50 μl reaction) of the *in vitro*–translated securin-CFP was added into each (15–20 µl) of the extracts with varying concentrations of Δ65–cyclin B1. After the extracts reached steady state levels of Cdk1 activity, they were loaded in a small volume 384-well black plate (Greiner, Germany), and the degradation of securin-CFP was monitored in real time using a fluorescence microplate reader (FLEXstation II 384). Duplicates or triplicates were typically performed.

## Supporting Information

Figure S1 **Raw images for quantitative Western blots and kinase assays, related to** **Figure 2** **.** (A–C) Linearity of Western blots and H1 kinase assays. Different amounts of egg lysate, expressed as egg-equivalents per lane, were subjected to Western blotting (for cyclin B1 and pY15–Cdk1) and H1 kinase assays (for Cdk1 activity). Bands were imaged using GelDoc and quantified using ImageJ. (D) The raw images of the cyclin B1 Western blots for Figure 2A. The four samples shown in the red box were duplicates used to normalize the intensities of the two blots. Peak cyclin B1 concentrations were higher in the first cycle than in subsequent cycles. (E) The raw images of pY15–Cdk1 Western blots for Figure 2A. The red box designates one duplicate sample used to normalize intensities. The blue box shows one interphase embryo from each of the first four cycles, loaded in consecutive wells. Peak levels of pY15–Cdk1 were higher in the first cycle than the subsequent cycles.
(TIF)

Figure S2 **pT161–Cdk1 levels correlate well with cyclin B1 levels, related to** **Figure 2** **.** (A) Raw images for quantitative Western blots for pT161–Cdk1 and cyclin B1. Samples taken from embryos in the first and second cycles are shown. The red box highlights samples of unfertilized eggs used as a titration series to check for linearity of the Western blot. For the embryos taken within the first cell cycle, the time labeled corresponds to time after fertilization. For the embryos taken within the second cell cycle, the time labeled corresponds to the time after the first division. (B) Correlation of cyclin B1 levels with pT161 levels. The same trend line fits data from interphase (cyclin B1 levels increasing, blue points) and M-phase (cyclin B1 levels decreasing, red points). (C) The same trend line also fits data from the first cycle (blue points) and the second and third cycles (red points). The correlation coefficient *r* ^2^  =  0.90.
(TIF)

Figure S3 **Activation of MAPK upon reentry of egg extracts, but not embryo extracts, into mitosis, related to** **Figure 4** **.** Interphase *Xenopus* egg and embryo extracts were prepared as described in Materials and Methods. One aliquot of the egg extract was treated with the MEK inhibitor U0126 (130 µM). Samples were collected at various times after taking the extracts off ice. The extracts reached interphase after 30 min at room temperature, at which point most of the endogenous cyclin B1 had been degraded (not shown). Δ65–cyclin B1 (50 nM) was then added to drive the extracts into mitosis, and samples were taken at various times for immunoblotting with a phospho-MAPK antibody.
(TIF)

Figure S4
**Estimating the reduction of the Wee1/Cdc25 ratio during the transition between the first cycle and the subsequent cycles, related to** **Figure 5** **.** (A, B) Inferring the change in *r* from the amplitude of pY15–Cdk1 oscillations. (A) Time courses of pY15–Cdk1 oscillations in the first four cell cycles, from two independent experiments. (A, inset) Modeled relationship between *r* and the amplitude of pY15–Cdk1 oscillations. (B) Approximate amplitude of pY15–Cdk1 oscillations as a function of cell cycle number. *r* falls by ∼8-fold. (C, D) The relationship between cyclin B1 levels and Cdk1 Y15 phosphorylation during the first four interphases. Assuming that Y15 phosphorylation equilibrates quickly relative to the changes in cyclin B1 levels, the relationship between pY15–Cdk1 and cyclin B1 is given by: 
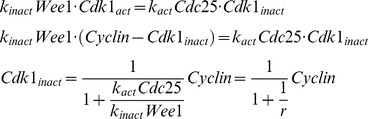
 Thus, when *r* is small, it is approximately given by the slope of the pY15–Cdk1 versus cyclin B1 plot (ΔpY15–Cdk1/Δcyclin B1). One plot showing cyclin B1 versus pY15–Cdk1 for two cell cycles is shown in (C). (D) Slope (ΔpY15–Cdk1/Δcyclin B1) versus cycle number. The slopes were normalized to the slope of the first cycle. The open circles represent the best fit values of the slopes, and the error bars correspond to one standard error. The dashed line corresponds to an 8-fold decrease of the slope, which is what we inferred from the pY15–Cdk1 amplitudes in (B), and assumed in our computational model of the transition between the first cycle and the subsequent cycles.(TIF)

Figure S5 **Modeled robustness and tunability in oscillators with strong and weak positive feedback, related to** **Figure 6** **.** (A, B) An alternative way of varying positive feedback strength. Here we varied the parameter *p*, which is the ratio of M-phase to interphase Cdc25 activity and interphase to M-phase Wee1 activity. We then calculated the steady state response of Cdk1 to cyclin B1 (A) and the robustness of the oscillations (B) for different assumed values of p (A, B) and different assumed levels of ultrasensitivity in the negative feedback loop (B). (C) Sensitivity analysis. Each parameter was varied ±10% from its nominal value, and the percentage change in the period was calculated. The values plotted are the absolute values of the sensitivities. The nominal values of r were 1/2 (purple) and 1/32 (red). (D) Period versus amplitude plots for the model with weak (top, red curves) or strong (bottom, blue curves) positive feedback. The black open circles represent the period and amplitude for the unperturbed model in its long period and short period regimes. The black curves show how the period and amplitude change as the assumed values of *r* and *ksynth* change. The red and blue curves show the calculated periods and amplitudes resulting from changes of each of the model’s other parameters. (E) Lack of tunability in the negative-feedback-only oscillator. As an example, the parameter tuned here is the APC inactivation rate constant *kapcoff*. There is a limited range of parameter values where oscillations occur. If cyclin degradation is turned off too rapidly (as shown in red), the cyclin would not be degraded sufficiently and the system reaches a steady state with high cyclin concentration. If cyclin degradation is turned off too slowly (as shown in blue), the cyclin in the system reaches a steady state with low cyclin concentration.
(TIF)

Figure S6 **Cyclin B1 synthesis in embryos treated with different concentrations of cycloheximide. (A) Cell cycle delay.** (B) Time course of cyclin accumulation. Note that a large decrease in cyclin B1 synthesis yields a relatively modest lengthening of the cell cycle. This is also true in our model of the embryonic cell cycle, as long as the change in *r* still occurs on schedule.
(TIF)

Movie S1 **Time-lapse movies of** ***Xenopus laevis*** **early embryonic cell cycles in PD0166285 and cycloheximide-treated embryos, related to** **Figure 7** **.** Embryos in the left upper quadrant were DMSO-treated control embryos. Embryos in the right upper quadrant were treated with 30 µM PD0166285 during the first cycle. Embryos in the left lower quadrant were treated with 0.25 µg/µl of cycloheximide during the first cycle. Embryos in the right lower quadrant were treated with 30 µM of PD0166285 and 0.25 µg/µl of cycloheximide during the first cycle. The movie starts at 2.5 h postfertilization and ends at 10.5 h postfertilization. Three snapshots are shown in Figure 7G.
(MOV)

Table S1 **Timing and variability of the first few embryonic cell cycles, related to** **Figure 1** **.**
(PDF)

Text S1 **Constructing a mathematical model for the** ***Xenopus laevis*** **embryonic cell cycle oscillator.**
(DOCX)

Text S2 **Random parameter selection to measure robustness of oscillations.**
(DOC)

## Abbreviations

APC/C: anaphase-promoting complex or cyclosome
CAK: Cdk-activating kinase
Cdk1: cyclin-dependent kinase 1
Ser: serine
Thr or T: threonine
Tyr or Y: tyrosine

## References

[1] Deppe U, Schierenberg E, Cole T, Krieg C, Schmitt D, et al (1978) Cell lineages of the embryo of the nematode Caenorhabditis elegans. Proc Natl Acad Sci U S A 75: 376–380. 272653 10.1073/pnas.75.1.376 PMC411251

[2] Foe VE, Odell GM, Edgar BA (1993) Mitosis and morphogenesis in the Drosphila embryo: point and counterpoint. In: Bate M, Martinez Arias A, editors. The development of Drosophila melanogaster. Cold Spring Harbor, NY: Cold Spring Harbor Laboratory Press. pp. 149–300.

[3] Masuda M, Sato H (1984) Asynchronization of cell division is concurrently related with ciliogenesis in sea urchin blastulae. Dev Growth Diff 26: 281–294. 10.1111/j.1440-169X.1984.00281.x 37281142

[4] Kimmel CB, Ballard WW, Kimmel SR, Ullmann B, Schilling TF (1995) Stages of embryonic development of the zebrafish. Dev Dyn 203: 253–310. 8589427 10.1002/aja.1002030302

[5] Kirschner MW, Gerhart JC (1981) Spatial and temporal changes in the amphibian egg. BioScience 31: 381–388.

[6] Hara K, Tydeman P, Kirschner M (1980) A cytoplasmic clock with the same period as the division cycle in Xenopus eggs. Proc Natl Acad Sci U S A 77: 462–466. 6928638 10.1073/pnas.77.1.462 PMC348291

[7] Murray AW, Kirschner MW (1989) Cyclin synthesis drives the early embryonic cell cycle. Nature 339: 275–280. 2566917 10.1038/339275a0

[8] Morgan DO (2007) The cell cycle: principles of control. London: New Science Press Ltd.

[9] Parker LL, Piwnica-Worms H (1992) Inactivation of the p34cdc2-cyclin B complex by the human WEE1 tyrosine kinase. Science 257: 1955–1957. 1384126 10.1126/science.1384126

[10] McGowan CH, Russell P (1993) Human Wee1 kinase inhibits cell division by phosphorylating p34cdc2 exclusively on Tyr15. Embo J 12: 75–85. 8428596 10.1002/j.1460-2075.1993.tb05633.x PMC413177

[11] Mueller PR, Coleman TR, Dunphy WG (1995) Cell cycle regulation of a Xenopus Wee1-like kinase. Mol Biol Cell 6: 119–134. 7749193 10.1091/mbc.6.1.119 PMC275819

[12] Tang Z, Coleman TR, Dunphy WG (1993) Two distinct mechanisms for negative regulation of the Wee1 protein kinase. Embo J 12: 3427–3436. 7504624 10.1002/j.1460-2075.1993.tb06017.x PMC413619

[13] Mueller PR, Coleman TR, Kumagai A, Dunphy WG (1995) Myt1: a membrane-associated inhibitory kinase that phosphorylates Cdc2 on both threonine-14 and tyrosine-15. Science 270: 86–90. 7569953 10.1126/science.270.5233.86

[14] Palmer A, Gavin AC, Nebreda AR (1998) A link between MAP kinase and p34^cdc2^/cyclin B during oocyte maturation: p90^rsk^ phosphorylates and inactivates the p34^cdc2^ inhibitory kinase Myt1. Embo J 17: 5037–5047. 9724639 10.1093/emboj/17.17.5037 PMC1170831

[15] Strausfeld U, Labbe JC, Fesquet D, Cavadore JC, Picard A, et al (1991) Dephosphorylation and activation of a p34cdc2/cyclin B complex in vitro by human CDC25 protein. Nature 351: 242–245. 1828290 10.1038/351242a0

[16] Kumagai A, Dunphy WG (1992) Regulation of the cdc25 protein during the cell cycle in Xenopus extracts. Cell 70: 139–151. 1623517 10.1016/0092-8674(92)90540-s

[17] Kim SH, Li C, Maller JL (1999) A maternal form of the phosphatase Cdc25A regulates early embryonic cell cycles in Xenopus laevis. Dev Biol 212: 381–391. 10433828 10.1006/dbio.1999.9361

[18] Millar JB, McGowan CH, Lenaers G, Jones R, Russell P (1991) p80cdc25 mitotic inducer is the tyrosine phosphatase that activates p34cdc2 kinase in fission yeast. EMBO J 10: 4301–4309. 1756737 10.1002/j.1460-2075.1991.tb05008.x PMC453183

[19] Hoffmann I, Clarke PR, Marcote MJ, Karsenti E, Draetta G (1993) Phosphorylation and activation of human cdc25-C by cdc2—cyclin B and its involvement in the self-amplification of MPF at mitosis. Embo J 12: 53–63. 8428594 10.1002/j.1460-2075.1993.tb05631.x PMC413175

[20] Solomon MJ, Glotzer M, Lee TH, Philippe M, Kirschner MW (1990) Cyclin activation of p34^cdc2^ . Cell 63: 1013–1024. 2147872 10.1016/0092-8674(90)90504-8

[21] Sha W, Moore J, Chen K, Lassaletta AD, Yi CS, et al (2003) Hysteresis drives cell-cycle transitions in Xenopus laevis egg extracts. Proc Natl Acad Sci U S A 100: 975–980. 12509509 10.1073/pnas.0235349100 PMC298711

[22] Pomerening JR, Sontag ED, Ferrell JE Jr (2003) Building a cell cycle oscillator: hysteresis and bistability in the activation of Cdc2. Nature Cell Biol 5: 346–351. 12629549 10.1038/ncb954

[23] Tsai TY, Choi YS, Ma W, Pomerening JR, Tang C, et al (2008) Robust, tunable biological oscillations from interlinked positive and negative feedback loops. Science 321: 126–129. 18599789 10.1126/science.1156951 PMC2728800

[24] Pomerening JR, Kim SY, Ferrell JE Jr (2005) Systems-level dissection of the cell-cycle oscillator: bypassing positive feedback produces damped oscillations. Cell 122: 565–578. 16122424 10.1016/j.cell.2005.06.016

[25] Lorca T, Castro A, Martinez AM, Vigneron S, Morin N, et al (1998) Fizzy is required for activation of the APC/cyclosome in Xenopus egg extracts. Embo J 17: 3565–3575. 9649427 10.1093/emboj/17.13.3565 PMC1170693

[26] Glotzer M, Murray AW, Kirschner MW (1991) Cyclin is degraded by the ubiquitin pathway. Nature 349: 132–138. 1846030 10.1038/349132a0

[27] Hartley RS, Rempel RE, Maller JL (1996) In vivo regulation of the early embryonic cell cycle in Xenopus. Dev Biol 173: 408–419. 8606001 10.1006/dbio.1996.0036

[28] Izumi T, Walker DH, Maller JL (1992) Periodic changes in phosphorylation of the Xenopus cdc25 phosphatase regulate its activity. Mol Biol Cell 3: 927–939. 1392080 10.1091/mbc.3.8.927 PMC275649

[29] Stanford JS, Ruderman JV (2005) Changes in regulatory phosphorylation of Cdc25C Ser287 and Wee1 Ser549 during normal cell cycle progression and checkpoint arrests. Mol Biol Cell 16: 5749–5760. 16195348 10.1091/mbc.E05-06-0541 PMC1289418

[30] Kumagai A, Yakowec PS, Dunphy WG (1998) 14-3-3 proteins act as negative regulators of the mitotic inducer Cdc25 in Xenopus egg extracts. Mol Biol Cell 9: 345–354. 9450960 10.1091/mbc.9.2.345 PMC25261

[31] Edgar BA, Sprenger F, Duronio RJ, Leopold P, O'Farrell PH (1994) Distinct molecular mechanism regulate cell cycle timing at successive stages of Drosophila embryogenesis. Genes Dev 8: 440–452. 7510257 10.1101/gad.8.4.440 PMC6520052

[32] Lolli G, Johnson LN (2005) CAK-Cyclin-dependent Activating Kinase: a key kinase in cell cycle control and a target for drugs? Cell Cycle 4: 572–577. 15876871

[33] Fisher RP (2005) Secrets of a double agent: CDK7 in cell-cycle control and transcription. J Cell Sci 118: 5171–5180. 16280550 10.1242/jcs.02718

[34] Solomon MJ, Lee T, Kirschner MW (1992) Role of phosphorylation in p34cdc2 activation: identification of an activating kinase. Mol Biol Cell 3: 13–27. 1532335 10.1091/mbc.3.1.13 PMC275499

[35] Tassan JP, Schultz SJ, Bartek J, Nigg EA (1994) Cell cycle analysis of the activity, subcellular localization, and subunit composition of human CAK (CDK-activating kinase). J Cell Biol 127: 467–478. 7929589 10.1083/jcb.127.2.467 PMC2120215

[36] Wang Y, Li J, Booher RN, Kraker A, Lawrence T, et al (2001) Radiosensitization of p53 mutant cells by PD0166285, a novel G(2) checkpoint abrogator. Cancer Res 61: 8211–8217. 11719452

[37] Potapova TA, Daum JR, Pittman BD, Hudson JR, Jones TN, et al (2006) The reversibility of mitotic exit in vertebrate cells. Nature 440: 954–958. 16612388 10.1038/nature04652 PMC1513549

[38] Lee G, White LS, Hurov KE, Stappenbeck TS, Piwnica-Worms H (2009) Response of small intestinal epithelial cells to acute disruption of cell division through CDC25 deletion. Proc Natl Acad Sci U S A 106: 4701–4706. 19273838 10.1073/pnas.0900751106 PMC2660721

[39] Murakami MS, Copeland TD, Vande Woude GF (1999) Mos positively regulates Xe-Wee1 to lengthen the first mitotic cell cycle of Xenopus. Genes Dev 13: 620–631. 10072389 10.1101/gad.13.5.620 PMC316506

[40] Walter SA, Guadagno SN, Ferrell JE Jr (2000) Activation of Wee1 by p42 MAPK in vitro and in cycling Xenopus egg extracts. Mol Biol Cell 11: 887–896. 10712507 10.1091/mbc.11.3.887 PMC14818

[41] Bitangcol JC, Chau AS, Stadnick E, Lohka MJ, Dicken B, et al (1998) Activation of the p42 mitogen-activated protein kinase pathway inhibits Cdc2 activation and entry into M-phase in cycling Xenopus egg extracts. Mol Biol Cell 9: 451–467. 9450967 10.1091/mbc.9.2.451 PMC25274

[42] Chesnel F, Vignaux F, Richard-Parpaillon L, Huguet A, Kubiak JZ (2005) Differences in regulation of the first two M-phases in Xenopus laevis embryo cell-free extracts. Dev Biol 285: 358–375. 16087172 10.1016/j.ydbio.2005.06.028

[43] Watanabe N, Hunt T, Ikawa Y, Sagata N (1991) Independent inactivation of MPF and cytostatic factor (Mos) upon fertilization of *Xenopus* eggs. Nature 352: 247–248. 1830371 10.1038/352247a0

[44] Yue J, Ferrell JE Jr (2004) Mos mediates the mitotic activation of p42 MAPK in Xenopus egg extracts. Curr Biol 14: 1581–1586. 15341746 10.1016/j.cub.2004.08.056

[45] Novak B, Tyson JJ (1993) Numerical analysis of a comprehensive model of M-phase control in Xenopus oocyte extracts and intact embryos. J Cell Sci 106: 1153–1168. 8126097 10.1242/jcs.106.4.1153

[46] Goldbeter A (1991) A minimal cascade model for the mitotic oscillator involving cyclin and cdc2 kinase. Proc Natl Acad Sci U S A 88: 9107–9111. 1833774 10.1073/pnas.88.20.9107 PMC52661

[47] Tyson JJ (1991) Modeling the cell division cycle: cdc2 and cyclin interactions. Proc Natl Acad Sci U S A 88: 7328–7332. 1831270 10.1073/pnas.88.16.7328 PMC52288

[48] Kim SY, Ferrell JE Jr (2007) Substrate competition as a source of ultrasensitivity in the inactivation of Wee1. Cell 128: 1133–1145. 17382882 10.1016/j.cell.2007.01.039

[49] Trunnell NB, Poon AC, Kim SY, Ferrell JE Jr (2011) Ultrasensitivity in the regulation of Cdc25C by Cdk1. Mol Cell 41: 263–274. 21292159 10.1016/j.molcel.2011.01.012 PMC3060667

[50] Yang Q, Ferrell JE Jr (2013) The Cdk1-APC/C cell cycle oscillator circuit functions as a time-delayed, ultrasensitive switch. Nat Cell Biol 15: 519–525. 23624406 10.1038/ncb2737 PMC3728279

[51] Labit H, Fujimitsu K, Bayin NS, Takaki T, Gannon J, et al (2012) Dephosphorylation of Cdc20 is required for its C-box-dependent activation of the APC/C. EMBO J 31: 3351–3362. 22713866 10.1038/emboj.2012.168 PMC3411074

[52] Vinod PK, Zhou X, Zhang T, Mayer TU, Novak B (2013) The role of APC/C inhibitor Emi2/XErp1 in oscillatory dynamics of early embryonic cell cycles. Biophys Chem 177–178: 1–6. 10.1016/j.bpc.2013.03.002 23562861

[53] Tischer T, Hormanseder E, Mayer TU (2012) The APC/C inhibitor XErp1/Emi2 is essential for Xenopus early embryonic divisions. Science 338: 520–524. 23019610 10.1126/science.1228394

[54] Ferrell JE Jr (2011) Simple rules for complex processes: new lessons from the budding yeast cell cycle. Mol Cell 43: 497–500. 21855788 10.1016/j.molcel.2011.08.002 PMC3160623

[55] Novak B, Tyson JJ (2008) Design principles of biochemical oscillators. Nat Rev Mol Cell Biol 9: 981–991. 18971947 10.1038/nrm2530 PMC2796343

[56] Ciemerych MA, Sicinski P (2005) Cell cycle in mouse development. Oncogene 24: 2877–2898. 15838522 10.1038/sj.onc.1208608

[57] Chen AA, Tan L, Suraj V, Reijo Pera R, Shen S (2013) Biomarkers identified with time-lapse imaging: discovery, validation, and practical application. Fertil Steril 99: 1035–1043. 23499001 10.1016/j.fertnstert.2013.01.143 PMC4283765

[58] Russell P, Nurse P (1986) cdc25^+^ functions as an inducer in the mitotic control of fission yeast. Cell 45: 145–153. 3955656 10.1016/0092-8674(86)90546-5

[59] Russell P, Nurse P (1987) Negative regulation of mitosis by wee1+, a gene encoding a protein kinase homolog. Cell 49: 559–567. 3032459 10.1016/0092-8674(87)90458-2

[60] Pomerening JR, Ubersax JA, Ferrell JE Jr (2008) Rapid cycling and precocious termination of G1 phase in cells expressing CDK1AF. Mol Biol Cell 19: 3426–3441. 18480403 10.1091/mbc.E08-02-0172 PMC2488275

[61] Booher RN, Deshaies RJ, Kirschner MW (1993) Properties of Saccharomyces cerevisiae wee1 and its differential regulation of p34CDC28 in response to G1 and G2 cyclins. EMBO J 12: 3417–3426. 8253069 10.1002/j.1460-2075.1993.tb06016.x PMC413617

[62] Coudreuse D, Nurse P (2010) Driving the cell cycle with a minimal CDK control network. Nature 468: 1074–1079. 21179163 10.1038/nature09543

[63] Schuster S, Marhl M, Hofer T (2002) Modelling of simple and complex calcium oscillations. From single-cell responses to intercellular signalling. Eur J Biochem 269: 1333–1355. 11874447 10.1046/j.0014-2956.2001.02720.x

[64] Goldbeter A (1995) A model for circadian oscillations in the Drosophila period protein (PER). Proc Biol Sci 261: 319–324. 8587874 10.1098/rspb.1995.0153

[65] Igoshin OA, Goldbeter A, Kaiser D, Oster G (2004) A biochemical oscillator explains several aspects of Myxococcus xanthus behavior during development. Proc Natl Acad Sci U S A 101: 15760–15765. 15496464 10.1073/pnas.0407111101 PMC524859

[66] Hoffmann A, Levchenko A, Scott ML, Baltimore D (2002) The IkappaB-NF-kappaB signaling module: temporal control and selective gene activation. Science 298: 1241–1245. 12424381 10.1126/science.1071914

[67] Murray AW (1991) Cell cycle extracts. Meth Cell Biol 36: 581–605. 1839804

[68] Smythe C, Newport JW (1991) Systems for the study of nuclear assembly, DNA replication, and nuclear breakdown in Xenopus laevis egg extracts. Methods Cell Biol 35: 449–468. 1664032 10.1016/s0091-679x(08)60583-x

[69] Hochegger H, Klotzbucher A, Kirk J, Howell M, le Guellec K, et al (2001) New B-type cyclin synthesis is required between meiosis I and II during Xenopus oocyte maturation. Development 128: 3795–3807. 11585805 10.1242/dev.128.19.3795

