# Supplementary figures and images for: Changes in Oscillatory Dynamics in the Cell Cycle of Early Xenopus laevis Embryos
Source: PLoS Biol. 2014 Feb 11;12(2):e1001788. doi: 10.1371/journal.pbio.1001788 (PMC3921120; doi:10.1371/journal.pbio.1001788)

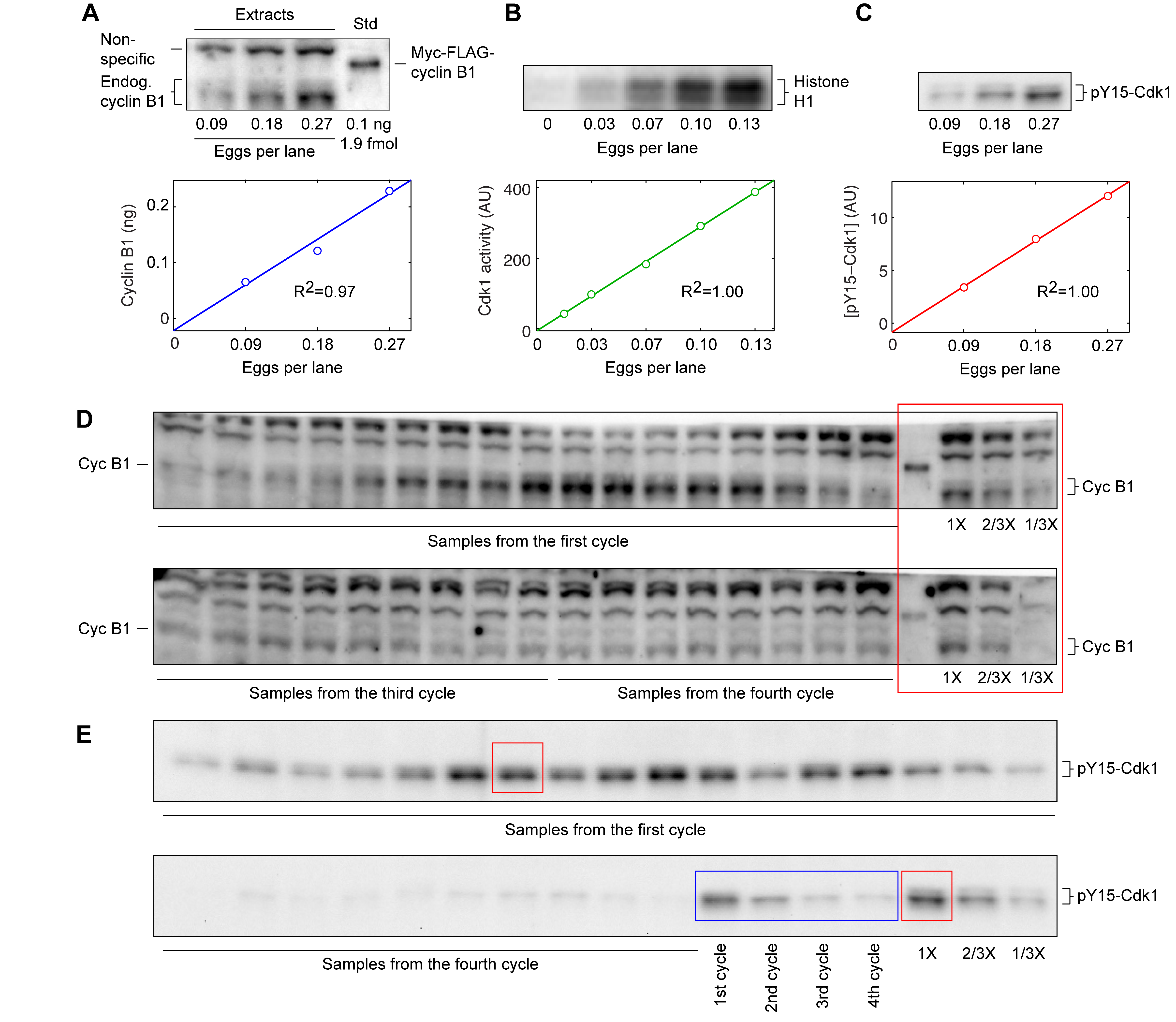

Supplement: Figure S1 — Raw images for quantitative Western blots and kinase assays, related to Figure 2 . (A–C) Linearity of Western blots and H1 kinase assays. Different amounts of egg lysate, expressed as egg-equivalents per lane, were subjected to Western blotting (for cyclin B1 and pY15–Cdk1) and H1 kinase assays (for Cdk1 activity). Bands were imaged using GelDoc and quantified using ImageJ. (D) The raw images of the cyclin B1 Western blots for Figure 2A. The four samples shown in the red box were duplicates used to normalize the intensities of the two blots. Peak cyclin B1 concentrations were higher in the first cycle than in subsequent cycles. (E) The raw images of pY15–Cdk1 Western blots for Figure 2A. The red box designates one duplicate sample used to normalize intensities. The blue box shows one interphase embryo from each of the first four cycles, loaded in consecutive wells. Peak levels of pY15–Cdk1 were higher in the first cycle than the subsequent cycles. (TIF) [file pbio.1001788.s001.tif]

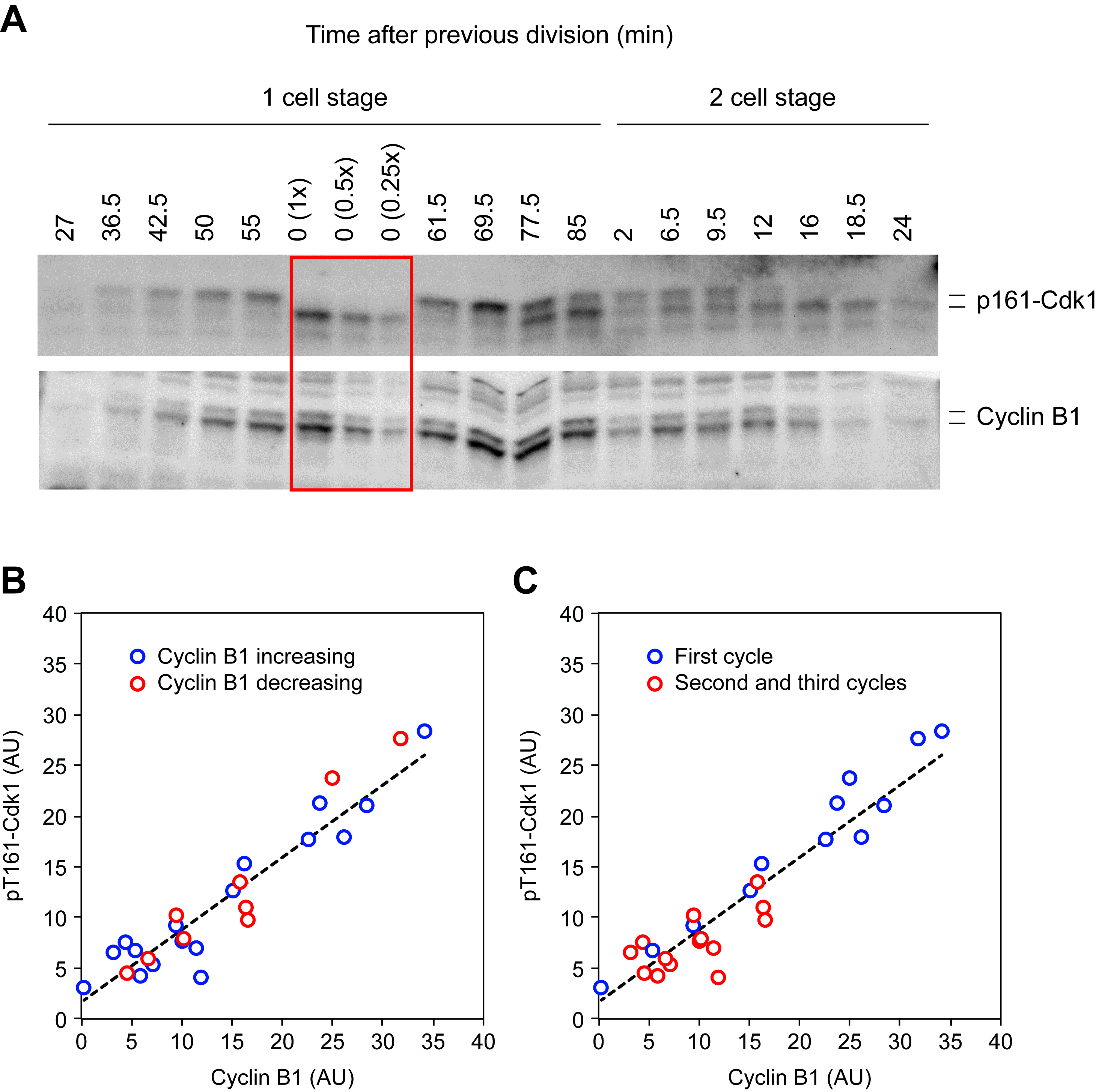

Supplement: Figure S2 — pT161–Cdk1 levels correlate well with cyclin B1 levels, related to Figure 2 . (A) Raw images for quantitative Western blots for pT161–Cdk1 and cyclin B1. Samples taken from embryos in the first and second cycles are shown. The red box highlights samples of unfertilized eggs used as a titration series to check for linearity of the Western blot. For the embryos taken within the first cell cycle, the time labeled corresponds to time after fertilization. For the embryos taken within the second cell cycle, the time labeled corresponds to the time after the first division. (B) Correlation of cyclin B1 levels with pT161 levels. The same trend line fits data from interphase (cyclin B1 levels increasing, blue points) and M-phase (cyclin B1 levels decreasing, red points). (C) The same trend line also fits data from the first cycle (blue points) and the second and third cycles (red points). The correlation coefficient r 2 = 0.90. (TIF) [file pbio.1001788.s002.tif]

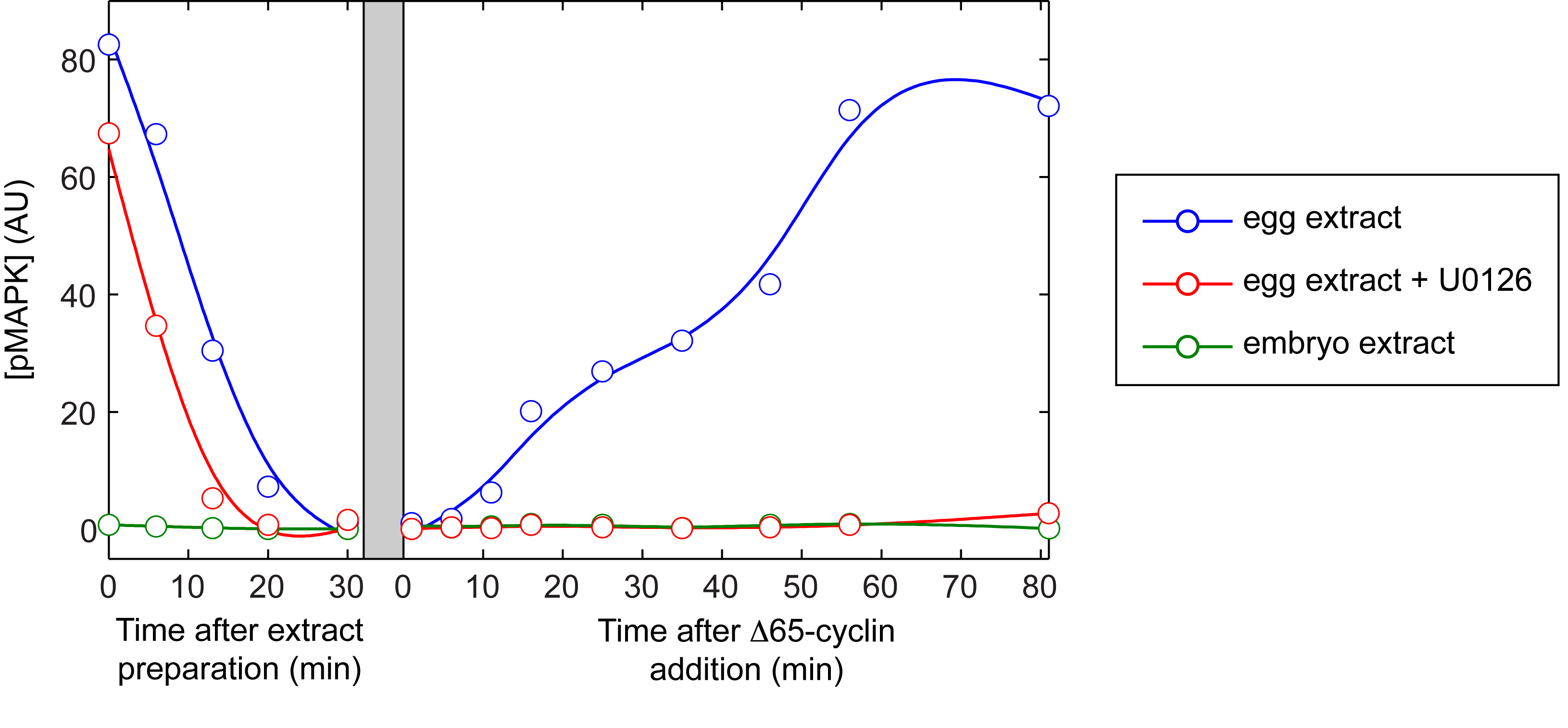

Supplement: Figure S3 — Activation of MAPK upon reentry of egg extracts, but not embryo extracts, into mitosis, related to Figure 4 . Interphase Xenopus egg and embryo extracts were prepared as described in Materials and Methods. One aliquot of the egg extract was treated with the MEK inhibitor U0126 (130 µM). Samples were collected at various times after taking the extracts off ice. The extracts reached interphase after 30 min at room temperature, at which point most of the endogenous cyclin B1 had been degraded (not shown). Δ65–cyclin B1 (50 nM) was then added to drive the extracts into mitosis, and samples were taken at various times for immunoblotting with a phospho-MAPK antibody. (TIF) [file pbio.1001788.s003.tif]

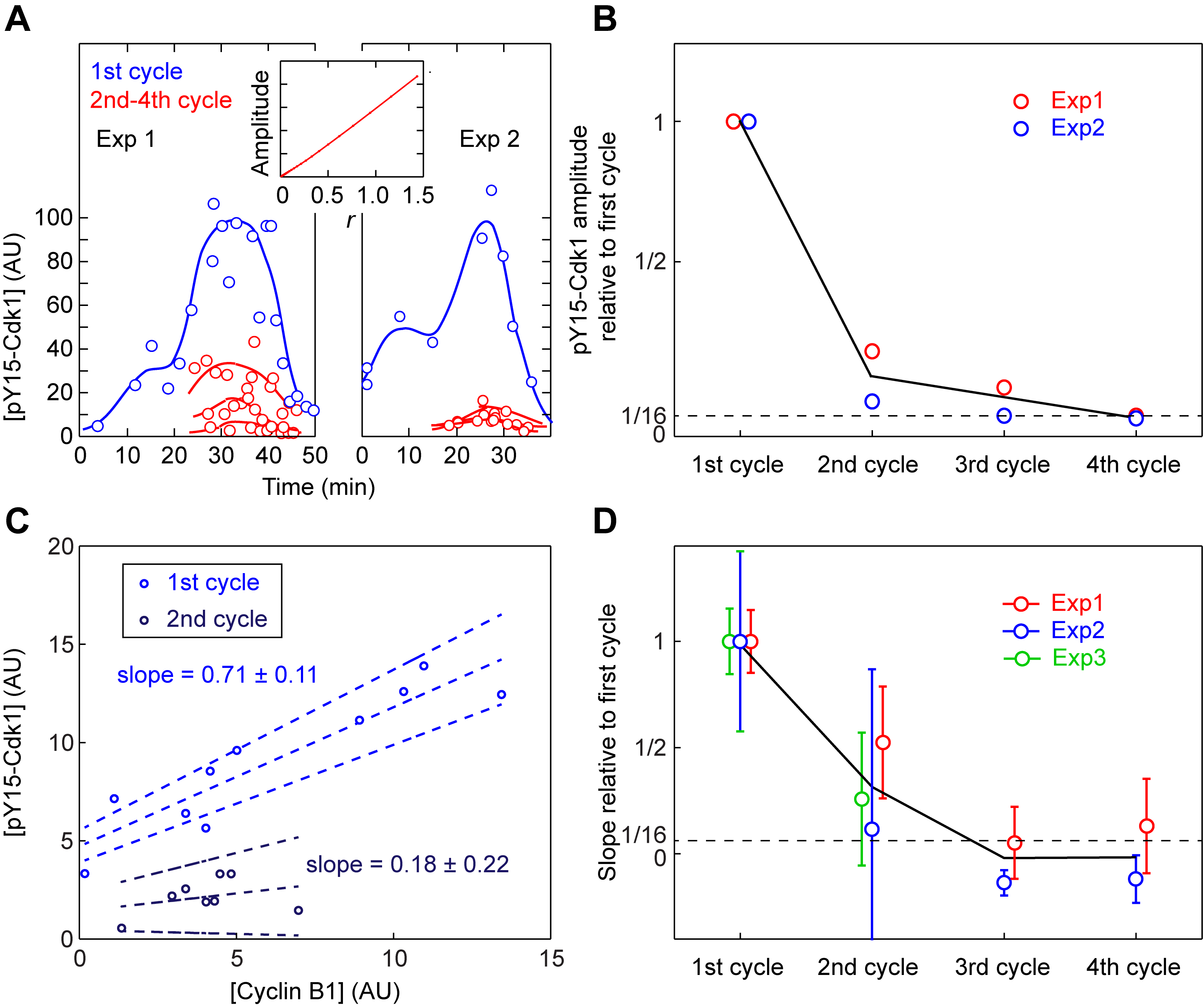

Supplement: Figure S4 — Estimating the reduction of the Wee1/Cdc25 ratio during the transition between the first cycle and the subsequent cycles, related to Figure 5 . (A, B) Inferring the change in r from the amplitude of pY15–Cdk1 oscillations. (A) Time courses of pY15–Cdk1 oscillations in the first four cell cycles, from two independent experiments. (A, inset) Modeled relationship between r and the amplitude of pY15–Cdk1 oscillations. (B) Approximate amplitude of pY15–Cdk1 oscillations as a function of cell cycle number. r falls by ∼8-fold. (C, D) The relationship between cyclin B1 levels and Cdk1 Y15 phosphorylation during the first four interphases. Assuming that Y15 phosphorylation equilibrates quickly relative to the changes in cyclin B1 levels, the relationship between pY15–Cdk1 and cyclin B1 is given by: Thus, when r is small, it is approximately given by the slope of the pY15–Cdk1 versus cyclin B1 plot (ΔpY15–Cdk1/Δcyclin B1). One plot showing cyclin B1 versus pY15–Cdk1 for two cell cycles is shown in (C). (D) Slope (ΔpY15–Cdk1/Δcyclin B1) versus cycle number. The slopes were normalized to the slope of the first cycle. The open circles represent the best fit values of the slopes, and the error bars correspond to one standard error. The dashed line corresponds to an 8-fold decrease of the slope, which is what we inferred from the pY15–Cdk1 amplitudes in (B), and assumed in our computational model of the transition between the first cycle and the subsequent cycles. (TIF) [file pbio.1001788.s004.tif]

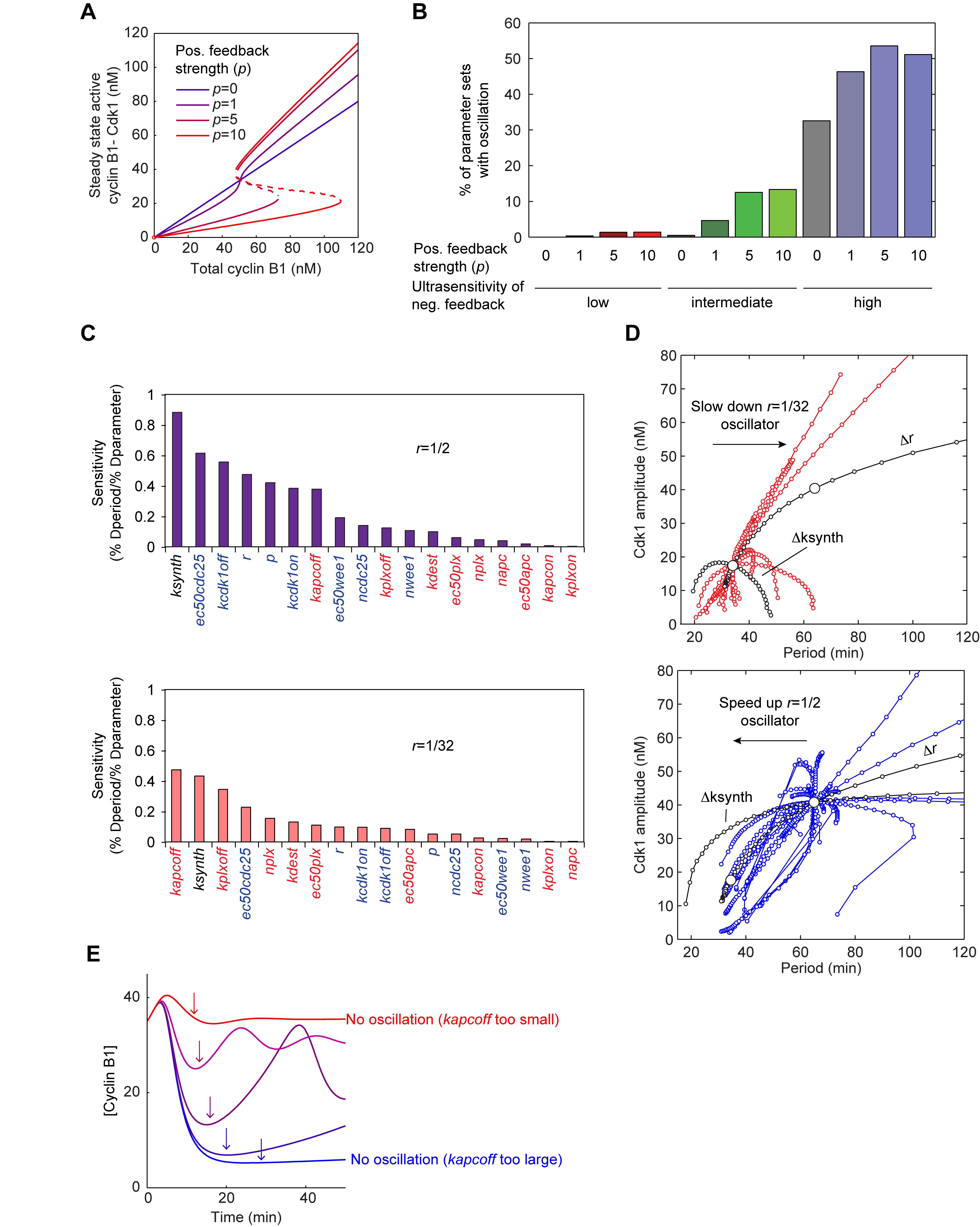

Supplement: Figure S5 — Modeled robustness and tunability in oscillators with strong and weak positive feedback, related to Figure 6 . (A, B) An alternative way of varying positive feedback strength. Here we varied the parameter p, which is the ratio of M-phase to interphase Cdc25 activity and interphase to M-phase Wee1 activity. We then calculated the steady state response of Cdk1 to cyclin B1 (A) and the robustness of the oscillations (B) for different assumed values of p (A, B) and different assumed levels of ultrasensitivity in the negative feedback loop (B). (C) Sensitivity analysis. Each parameter was varied ±10% from its nominal value, and the percentage change in the period was calculated. The values plotted are the absolute values of the sensitivities. The nominal values of r were 1/2 (purple) and 1/32 (red). (D) Period versus amplitude plots for the model with weak (top, red curves) or strong (bottom, blue curves) positive feedback. The black open circles represent the period and amplitude for the unperturbed model in its long period and short period regimes. The black curves show how the period and amplitude change as the assumed values of r and ksynth change. The red and blue curves show the calculated periods and amplitudes resulting from changes of each of the model’s other parameters. (E) Lack of tunability in the negative-feedback-only oscillator. As an example, the parameter tuned here is the APC inactivation rate constant kapcoff. There is a limited range of parameter values where oscillations occur. If cyclin degradation is turned off too rapidly (as shown in red), the cyclin would not be degraded sufficiently and the system reaches a steady state with high cyclin concentration. If cyclin degradation is turned off too slowly (as shown in blue), the cyclin in the system reaches a steady state with low cyclin concentration. (TIF) [file pbio.1001788.s005.tif]

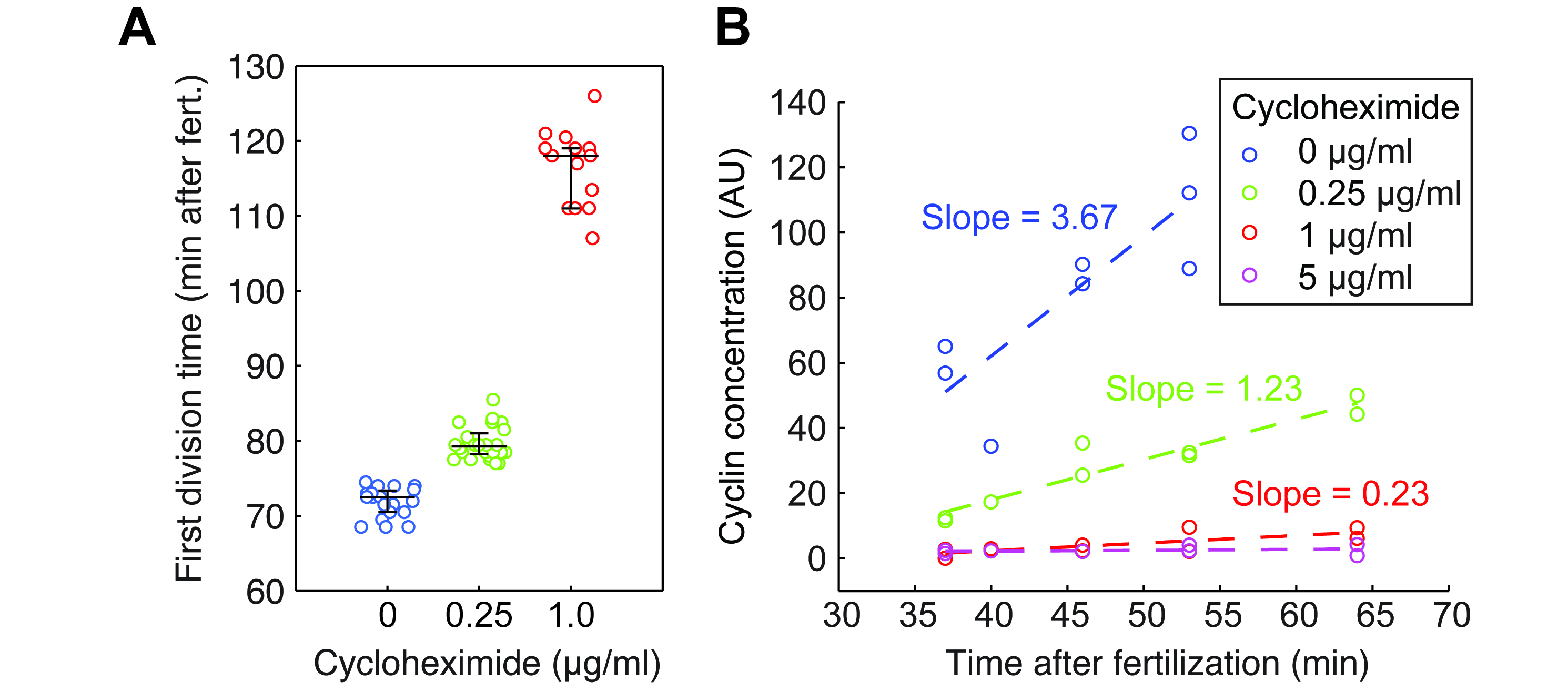

Supplement: Figure S6 — Cyclin B1 synthesis in embryos treated with different concentrations of cycloheximide. (A) Cell cycle delay. (B) Time course of cyclin accumulation. Note that a large decrease in cyclin B1 synthesis yields a relatively modest lengthening of the cell cycle. This is also true in our model of the embryonic cell cycle, as long as the change in r still occurs on schedule. (TIF) [file pbio.1001788.s006.tif]
