# Supplementary material for: Changes in Oscillatory Dynamics in the Cell Cycle of Early Xenopus laevis Embryos
Source: PLoS Biol. 2014 Feb 11;12(2):e1001788. doi: 10.1371/journal.pbio.1001788 (PMC3921120; doi:10.1371/journal.pbio.1001788)
Supplement: Table S1 — Timing and variability of the first few embryonic cell cycles, related to Figure 1 . (PDF) [file pbio.1001788.s008.pdf]

Table S1. Timing and Variability of the First Few Embryonic Cell Cycles, Related to Figure 1

	Cycle 1 (fertilization to division)	Cycle 1 (rotation to division)	Cycle 2	Cycle 3	Cycle 4
Experiment 1 (n=88)					
Mean period	86.5 min	74.0 min	29.2 min	29.4 min	n.d.
S.D.	2.7 min	3.1 min	1.3 min	1.1 min	n.d.
S.D./period	0.031	0.042	0.043	0.037	n.d.
Experiment 2 (n=71)					
Mean period	93.4 min	80.9 min	31.8 min	29.8 min	28.5 min
S.D.	5.4 min	4.9 min	2.3 min	2.3 min	1.7 min
S.D./Period	0.058	0.061	0.071	0.076	0.074

Variability is expressed as standard deviation (S.D.) and coefficient of variation (S.D./period). Temperature in both experiments was 23°.
