# Supplementary material for: Changes in Oscillatory Dynamics in the Cell Cycle of Early Xenopus laevis Embryos
Source: PLoS Biol. 2014 Feb 11;12(2):e1001788. doi: 10.1371/journal.pbio.1001788 (PMC3921120; doi:10.1371/journal.pbio.1001788)
Supplement: Text S1 — Constructing a mathematical model for the Xenopus laevis embryonic cell cycle oscillator. (DOCX) [file pbio.1001788.s009.docx]

**Text S1**

**Constructing a mathematical model for the *Xenopus laevis* embryonic cell cycle oscillator**

Previously we have used relatively complicated ODE models, with many undetermined parameters, to describe the *Xenopus* embryonic cell cycle [[1](#_ENREF_1),[2](#_ENREF_2)]. Recently we have been using simpler models [[3](#_ENREF_3),[4](#_ENREF_4)]. The rationale for using simpler models is, first, that they display very similar behaviors to the more complicated models; second, that they reproduce the observed cycles of cyclin abundance and Cdk1 activation about as well as the complicated models do; and third, that they contain fewer parameters, many of which have now been experimentally determined (see below). Our simple models are conceptually similar to the first cell cycle models proposed by Novak and Tyson [[5](#_ENREF_5),[6](#_ENREF_6)], although the functional forms and parameters used are different.

We started by writing equations for the synthesis and destruction of cyclin B and the activation and inactivation of cyclin B-Cdk1 complexes. We assumed that cyclin B was synthesized at a constant rate, and that it quickly bound to Cdk1, which was assumed to be present in excess, and was quickly phosphorylated by constitutively-active CAK. These assumptions meant that cyclin synthesis initially produced active cyclin B-Cdk1. We then assumed the these complexes could be inactivated by Wee1 or destroyed by APC/C and the proteasome, and that active cyclin B1-Cdk1 could be produced from inactive cyclin B1-Cdk1 by Cdc25. We assumed mass action kinetics for all of these processes. This yielded two ODEs:

$$\begin{matrix} \frac{d[cdk1_{a}]}{dt}=k_{synth}-k_{dest}[apc_{a}][cdk1_{a}]+k_{cdk1on}[cdc25_{a}][cdk1_{i}]-k_{cdk1off}[wee1_{a}][cdk1_{a}] \\ \frac{d[cdk1_{i}]}{dt}=-k_{dest}[apc_{a}][cdk1_{i}]-k_{cdk1on}[cdc25_{a}][cdk1_{i}]+k_{cdk1off}[wee1_{a}][cdk1_{a}] \end{matrix}$$

Next we assumed that the regulation of Cdc25 and Wee1 by Cdk1 (and some unspecified phosphatase) was rapid, allowing us to replace the variables *cdc25_a_* and *weel_a_* with functions of Cdk1. Experimental studies have shown that these three response functions can all be approximated by Hill functions with high Hill exponents. It follows that:

$$\begin{matrix} \frac{d[cdk1_{a}]}{dt}=k_{synth}-k_{dest}[apc_{a}][cdk1_{a}]+k_{cdk1on}\left(1+p\frac{[cdk1_{a}]^{ncdc25}}{[cdk1_{a}]^{ncdc25}+ec50_{cdc25}^{ncdc25}}\right)[cdk1_{i}] \\ -k_{cdk1off}\left(1+p\frac{ec50_{wee1}^{nwee1}}{[cdk1_{a}]^{nwee1}+ec50_{wee1}^{nwee1}}\right)[cdk1_{a}] \\ \frac{d[cdk1_{i}]}{dt}=-k_{dest}[apc_{a}][cdk1_{i}]-k_{cdk1on}\left(1+p\frac{[cdk1_{a}]^{ncdc25}}{[cdk1_{a}]^{ncdc25}+ec50_{cdc25}^{ncdc25}}\right)[cdk1_{i}] \\ +k_{cdk1off}\left(1+p\frac{ec50_{wee1}^{nwee1}}{[cdk1_{a}]^{nwee1}+ec50_{wee1}^{nwee1}}\right)[cdk1_{a}] \end{matrix}$$

The parameter *p* defines the activity of maximally-activated Cdc25 and Wee1 relative to their basal activities. For both proteins, the value has been experimentally estimated to be ~5 [[7-9](#_ENREF_7)].

Since Wee1 specific activity appears to decrease between the first cycle and the second, and Cdc25 abundance increases as Cdc25A accumulates, we introduce a parameter *r* that allows the ratio of the rates of Cdk1 activation and inactivation to be adjusted. For simplicity we assumed that Cdc25 and Wee1 each changed by the same factor, $\sqrt{r}$:

$$\begin{matrix} \frac{d[cdk1_{a}]}{dt}=k_{synth}-k_{dest}[apc_{a}][cdk1_{a}]+\frac{1}{\sqrt{r}}k_{cdk1on}\left(1+p\frac{[cdk1_{a}]^{ncdc25}}{[cdk1_{a}]^{ncdc25}+ec50_{cdc25}^{ncdc25}}\right)[cdk1_{i}] \\ -\sqrt{r}k_{cdk1off}\left(1+p\frac{ec50_{wee1}^{nwee1}}{[cdk1_{a}]^{nwee1}+ec50_{wee1}^{nwee1}}\right)[cdk1_{a}] \\ \frac{d[cdk1_{i}]}{dt}=-k_{dest}[apc_{a}][cdk1_{i}]-\frac{1}{\sqrt{r}}k_{cdk1on}\left(1+p\frac{[cdk1_{a}]^{ncdc25}}{[cdk1_{a}]^{ncdc25}+ec50_{cdc25}^{ncdc25}}\right)[cdk1_{i}] \\ +\sqrt{r}k_{cdk1off}\left(1+p\frac{ec50_{wee1}^{nwee1}}{[cdk1_{a}]^{nwee1}+ec50_{wee1}^{nwee1}}\right)[cdk1_{a}] \end{matrix}$$

Finally, we assumed that the change in *r* was described by a sigmoidal function of the form:

$$r(t)=\frac{1}{2\left(1+\frac{3}{1+\mathrm{e}^{(\frac{t_{0}-t}{\tau})}}\right)^{2}}$$

The activation of APC/C by Cdk1 is switch-like with a time delay [[3](#_ENREF_3)]. To reproduce both of these aspects of APC/C regulation in a simple ODE framework, we assumed that APC/C was activated in a two step process, with both steps involving highly ultrasensitive response functions. The first step was taken to be Plx1 activation; the second, APC/C activation. This yielded a four ODE model of the cell cycle where the ratio of Wee1 to Cdc25 activity could be varied as desired:

Note though that there are other plausible ways of implementing a delay [[3](#_ENREF_3),[10-12](#_ENREF_10)].

For initial conditions we chose:

$$\begin{matrix} cdk1_{a}[0]=60 \text{nM} \\ cdk1_{i}[0]=0 \text{nM} \\ plx_{a}[0]=0 \\ apc_{a}[0]=0 \end{matrix}$$

The rationale behind the choices of response functions and parameters is described below.

**Cyclin synthesis and degradation**

The quantitative western blotting results in Figure 2A and 2B provided a good reference for estimating the rates of synthesis and degradation of cyclin. We estimated the cyclin synthesis rate in both the first cycle and the subsequent cycles to be 1.5 nM/min, based on the observation that cyclin increased by about 75 nM in 50 min in the first cycle, and increased by about 25 nM in 15 min in the subsequent cycles. This value assumed that degradation is negligible during interphase. We estimated the degradation rate of cyclin to be 0.4 min^-1^. This is based on the observation that [cyclin B1] dropped to 1/8 its peak value within 15 minutes in Figure 2A. If the half-life is 5 minutes, then the estimated degradation rate $k_{dest}=\frac{\ln(2)}{t_{1/2}}=\frac{0.693}{5\min}=0.14\min^{-1}$. However, this assumed that the degradation machinery is fully active (i.e., [apc_a_]=1) during the whole time when cyclin is degraded. Since APC activation is not instantaneous [[3](#_ENREF_3)], we chose a higher value for *k_dest_* = 0.4 min^-1^. We also assumed a two-step model for APC/C^Cdc20^ activation to introduce a time lag into the process. Taken together, the model fit the experimental data well.

| Parameter | Value | Meaning | Justification |
| --- | --- | --- | --- |
| $k_{synth}$ | 1.5 nM/min | Synthesis rate of cyclin B1 | Estimated from Figure 2A |
| $k_{dest}$ | 0.4 | Degradation rate of cyclin B1 by active APC/C^Cdc20^ | Estimated from Figures 2A and B |

**Positive feedback loops**

The activation of Cdc25 and inactivation of Wee1 in response to Cdk1 activity have been measured quantitatively using *Xenopus* egg extracts. Therefore, both equation (1) and (2) are entirely based on experimental measurements. The parameter values correspond to the positive feedback [[13-15](#_ENREF_13)] are:

| Parameter | Value | Meaning | Justification |
| --- | --- | --- | --- |
| $ec50_{cdc25}$ | 30 nM | EC50 for the response of Cdc25 to Cdk1 | [[14](#_ENREF_14)] |
| $n_{cdc25}$ | 11 | Hill exponent for the response of Cdc25 to Cdk1 | [[14](#_ENREF_14)] |
| $ec50_{wee1}$ | 35 nM | EC50 for the response of Wee1 to Cdk1 | [[13](#_ENREF_13)] |
| $n_{wee1}$ | 3.5 | Hill exponent for the response of Wee1 to Cdk1 | [[13](#_ENREF_13)] |
| $k_{cdk1on}$ | 0.0354 | $k_{cdk1on}(1+p)/\sqrt{r}$ is the maximal rate of pY15-Cdk1 dephosphorylation | We expect this dephosphorylation rate $k_{cdk1on}(1+p)/\sqrt{r}=6\sqrt{2}k_{cdk1on}$ to be faster than the rate of cyclin synthesis, because of the switch-like activation of the Cdk1 activity in the first cycle. |
| $k_{cdk1off}$ | 0.0354 | $k_{cdk1off}(1+p)\sqrt{r}$ is the maximal rate of Y15-Cdk1 phosphorylation | We expect the phosphorylation rate $k_{cdk1off}(1+p)\sqrt{r}=3\sqrt{2}k_{cdk1off}$to be faster than the synthesis rate of cyclin because we observe good temporal correlation between cyclin concentration and pY15-Cdk1 level. In addition, we observe very little increase in Cdk1 activity during the first interphase. Therefore, we speculate that the maximal phosphorylation rate $k_{cdk1off}(1+p)\sqrt{r}=3\sqrt{2}k_{cdk1off}$should be much greater than the basal dephosphorylation rate $k_{cdk1on}$ during interphase. |
| *p* | 5 | The ratio  of $\frac{Wee1_{active}}{Wee1_{basal}}$and $\frac{Cdc25_{active}}{Cdc25_{basal}}$ | This parameter means active Wee1 is *p* times more active than its basal activity, and active Cdc25 is *p* times more active than its basal activity. We estimate this value to be 5 from previous measurements. [[7](#_ENREF_7),[16-18](#_ENREF_16)] |

**The Wee1-to-Cdc25 ratio (*r*) in the first and subsequent cycles , and the transition control parameter τ and t0**

We introduced the Wee1 to Cdc25 ratio (*r*) to tune the strength of the positive feedback. In the manuscript, we used the experimentally-measured steady-state relationship between cyclin B1 concentration and Cdk1 activity [[15](#_ENREF_15)] to calibrate this value. We determined that an *r* value between 0.25 and 0.5 best matched the dose/response curves for egg extracts (Figure 5D), which corresponded to the cytoplasmic environment of the first cell cycle. To model the first cycle, we started with *r* at a value of 0.5, and assumed a decrease in the value of *r* over time in the first cycle. We put in a control parameter t0 and τ. t0 represents the time that the transition from a first-cycle parameter to a subsequent-cycle parameter takes place. τ represents the smoothness of this transition. When the embryo is entering the subsequent cycles, the *r* value decreases to 1/32, a value we estimated to be most similar to the subsequent cycle environment.

**The negative feedback loop**

The onset of cyclin degradation has been shown to be ultrasensitive, with a ~15 min time delay in response of APC/C^Cdc20^ to Cdk1 [[3](#_ENREF_3)]. This can be modeled by either delay differential equations or by a system of coupled ODEs. The delay differential equation assume the delay times for APC/C^Cdc20^ activation and inactivation are the same. But we do not have evidence that this is true. Therefore, we chose to model the negative feedback as a set of ODEs and assumed that the activation of APC/C^Cdc20^ could be described as a two-step reaction for simplicity. We assumed that Cdk1 activates Plx1, and Plx1 activates APC/C^Cdc20^. These two steps can be viewed as a coarse-grained representation of the whole activation pathway from Cdk1 to APC/C^Cdc20^. How Cdk1 activates the degradation pathway is quantitatively measured. Since we break this into a 2-step reaction, the detailed parameters in each of the two steps are estimated with the goal to reproduce the quantitative measurement of the single dose response curve from Cdk1 activity to APC/C^Cdc20^ activity.

To match the EC50 of the degradation reaction with the experimentally measured value, some mathematical transformation is needed. In steady state:

$$\begin{matrix} plx_{a}=\frac{k_{plxon}plx_{tot}cdk1_{a}^{n_{plx}}}{(k_{plxon}+k_{plxoff})cdk1_{a}^{n_{plx}}+k_{plxoff}ec50_{plx}^{n_{plx}}} \\ =\frac{k_{plxon}plx_{tot}}{(k_{plxon}+k_{plxoff})}\frac{cdk1_{a}^{n_{plx}}}{cdk1_{a}^{n_{plx}}+\{[\frac{k_{plxoff}}{(k_{plxon}+k_{plxoff})}]^{\frac{1}{n_{plx}}}ec50_{plx}\}^{n_{plx}}} \end{matrix}$$

Therefore, the apparent EC50 of plx in response to Cdk1 is $[\frac{k_{plxoff}}{(k_{plxon}+k_{plxoff})}]^{\frac{1}{n_{plx}}}ec50_{plx}\equiv ec50_{plx}^{*}$

If we assumed *k_plxoff_* to be about 1/10 of *k_plxon_* and *n_plx_* about 5, then $ec50_{plx}^{*}$ is about 0.6**ec50_plx_*. The experimentally measured value of the EC50 is about the same as the EC50 for wee1 and cdc25, which are 30 – 35 nM [[3](#_ENREF_3)]. Therefore, we estimate the ec50_plx_ as 60 nM, with the apparent EC50 ec50_plx_^*^ ~ 36 nM.

Similarly for APC/C^Cdc20^:

The apparent Hill coefficient is roughly the product of the Hill coefficient of the two steps, which is *n_plx_* * *n_apc_*. We assigned *n_plx_* =5 and *n_apc_* =4 to obtain a compounded Hill coefficient of 20, which is similar to the experimentally measured Hill coefficient of the degradation reaction in *Xenopus laevis* extracts [[3](#_ENREF_3)].

| Parameter | Value | Meaning | Justification |
| --- | --- | --- | --- |
| $ec50_{plx}$ | 60 nM | EC50 of the dose response curve for Cdk1 to activate Plx1 | The EC50 for Cdk1 to activate cyclin degradation is only slightly larger than the EC50 for Cdk1 to phosphorylate Wee1 and Cdc25 [[3](#_ENREF_3)] |
| $ec50_{apc}$ | 0.5 | Non-dimensionalized EC50 for the response of APC/C^Cdc20^ to Plx1 | [[3](#_ENREF_3)] |
| $n_{apc}$ | 4 | Hill exponent for the response of APC/C^Cdc20^ to Plx1 | [[3](#_ENREF_3)] |
| $n_{plx}$ | 5 | Hill exponent for the response of Plx1 to Cdk1 | [[3](#_ENREF_3)] |
| $k_{plxon}$ | 1.5 | The rate of activation of Plx1 by active Cdk1 | Estimated assuming fast activation of Plx1 once Cdk1 activity passes a threshold |
| $k_{plxoff}$ | 0.125 | The rate of inactivation of Plx1 after Cdk1 is inactivated | Estimated based on the duration of mitotic exit |
| $k_{apcon}$ | 1.5 | The rate of activation of APC/C^Cdc20^ by active Plx1 | Estimated assuming fast activation of APC/C^Cdc20^ once Cdk1 activity passes a threshold |
| $k_{apcoff}$ | 0.15 | The rate of inactivation of APC/C^Cdc20^ after Plx1 is inactivated | Estimated based on the duration of mitotic exit |

We did not have direct measurement of when the APC/C^Cdc20^ becomes active when Cdk1 activity exceeds the threshold to activate APC/C^Cdc20^. We assumed this step to be fast and arbitrarily chose a value of 1.5 min^-1^ for both the activation of Plx and APC/C.

We then tried to decide the rate to switch off the degradation after Cdk1 level falls below the threshold to activate APC/C. For simplicity, we modeled the inactivation of Plx and APC/C^Cdc20^ as first order reactions :

Inactivation rate of [plx_a_] = $-k_{plxoff}[plx_{a}]$

Inactivation rate of [apc_a_] = $-k_{apcoff}[apc_{a}]$

The time it takes to turn off the degradation after mitotic exit depends on two rate constants *k_plxoff_* and *k_apcoff_*. Specifically, the half-life of Plx is $\frac{\ln2}{k_{plxoff}}$and the half-life of APC/C^Cdc20^ is $\frac{\ln2}{k_{apcoff}}$. In our parameter selection, *k_plxoff_* was chosen to be 0.15 nd *k_apcoff_* was chosen to be 0.125. This means the half-life of Plx and APC/C are about 5 minutes. We have assumed the amount of Cdk1 to turn on 50% of Plx is 36nM (as shown in the previous paragraph), and the fraction of active Plx to turn on 50% of APC/C is 0.5. This implies less than 50% of the Plx would remain active 5 min after Cdk1 level falls below 36 nM, and less than 50% of the APC/C would remain active 10 min after Cdk1 level falls below 36 nM. In Figures 2A and 2B, the duration in which [cyclin B1] was decreasing is about 15 min. This is consistent with the values chosen for *k_plxoff_* and *k_apcoff._*

**Supplemental References**

1. Pomerening JR, Sontag ED, Ferrell JE, Jr. (2003) Building a cell cycle oscillator: hysteresis and bistability in the activation of Cdc2. Nature Cell Biol 5: 346-351.

2. Tsai TY, Choi YS, Ma W, Pomerening JR, Tang C, et al. (2008) Robust, tunable biological oscillations from interlinked positive and negative feedback loops. Science 321: 126-129.

3. Yang Q, Ferrell JE, Jr. (2013) The Cdk1-APC/C cell cycle oscillator circuit functions as a time-delayed, ultrasensitive switch. Nat Cell Biol 15: 519-525.

4. Chang JB, Ferrell JE, Jr. (2013) Mitotic trigger waves and the spatial coordination of the Xenopus cell cycle. Nature 500: 603-607.

5. Novak B, Tyson JJ (1993) Numerical analysis of a comprehensive model of M-phase control in Xenopus oocyte extracts and intact embryos. J Cell Sci 106: 1153-1168.

6. Novak B, Tyson JJ (1993) Modeling the cell division cycle: M-phase trigger, oscillations, and size control. J Theor Biol 165: 101-134.

7. Kumagai A, Dunphy WG (1992) Regulation of the cdc25 protein during the cell cycle in Xenopus extracts. Cell 70: 139-151.

8. Mueller PR, Coleman TR, Dunphy WG (1995) Cell cycle regulation of a Xenopus Wee1-like kinase. Mol Biol Cell 6: 119-134.

9. Kim SY, Song EJ, Lee KJ, Ferrell JE, Jr. (2005) Multisite M-phase phosphorylation of Xenopus Wee1A. Mol Cell Biol 25: 10580-10590.

10. Tischer T, Hormanseder E, Mayer TU (2012) The APC/C inhibitor XErp1/Emi2 is essential for Xenopus early embryonic divisions. Science 338: 520-524.

11. Vinod PK, Zhou X, Zhang T, Mayer TU, Novak B (2013) The role of APC/C inhibitor Emi2/XErp1 in oscillatory dynamics of early embryonic cell cycles. Biophys Chem 177-178: 1-6.

12. Labit H, Fujimitsu K, Bayin NS, Takaki T, Gannon J, et al. (2012) Dephosphorylation of Cdc20 is required for its C-box-dependent activation of the APC/C. EMBO J 31: 3351-3362.

13. Kim SY, Ferrell JE, Jr. (2007) Substrate competition as a source of ultrasensitivity in the inactivation of Wee1. Cell 128: 1133-1145.

14. Trunnell NB, Poon AC, Kim SY, Ferrell JE, Jr. (2011) Ultrasensitivity in the regulation of Cdc25C by Cdk1. Mol Cell 41: 263-274.

15. Pomerening JR, Sontag ED, Ferrell JE, Jr. (2003) Building a cell cycle oscillator: hysteresis and bistability in the activation of Cdc2. Nat Cell Biol 5: 346-351.

16. Mueller PR, Coleman TR, Dunphy WG (1995) Cell cycle regulation of a Xenopus Wee1-like kinase. Mol Biol Cell 6: 119-134.

17. Kim SY, Song EJ, Lee KJ, Ferrell JE, Jr. (2005) Multisite M-Phase phosphorylation of Xenopus Wee1A. Mol Cell Biol 25: 10580-10590.

18. Tsai TY-C, Choi YS, Ma W, Pomerening JR, Tang C, et al. (2008) Robust, tunable biological oscillations from interlinked positive and negative feedback loops. Science 321: 126-129.
