# Supplementary material for: Changes in Oscillatory Dynamics in the Cell Cycle of Early Xenopus laevis Embryos
Source: PLoS Biol. 2014 Feb 11;12(2):e1001788. doi: 10.1371/journal.pbio.1001788 (PMC3921120; doi:10.1371/journal.pbio.1001788)
Supplement: Text S2 — Random parameter selection to measure robustness of oscillations. (DOC) [file pbio.1001788.s010.doc]

**Text S2**

**Random Parameter Selection to Measure Robustness of Oscillations**

To measure the likelihood of a given circuit topology to exhibit sustained oscillation, we applied random parameter selection to our ODE model and determined the percentage of random parameter sets that produced sustained oscillation. We were especially interested in comparing the topology with strong positive feedback versus weak positive feedback, and also the topology with high ultrasensitivity of negative feedback versus low ultrasensitivity of negative feedback. Therefore, we fixed the *r* value for a given strength of the positive feedback, and we fixed the Hill coefficient of *n_plx_* and *n_apc_*. As shown in Figure 6A, we created a spectrum of positive feedback strength by fixing the *r* value to be 1/2, 1/4, 1/8, and 1/32. For different ultrasensitivity of negative feedback, we fixed the Hill coefficients (*n_plx_* , *n_apc_*) to be (2,2), (3,3), and (6,6) for low, medium, and high ultrasensitivity of negative feedback. These parameters (*r*, *n_plx_*, *n_apc_*) were fixed throughout the random parameter selection process.

For the remaining parameters, we started with the value that best fit our data (shown in Figure 5F). For the parameters measured experimentally (such as the EC50 values and Hill exponents for the responses of Cdc25 and Wee1 to Cdk1), we chose a range that was roughly the measured value ± 50%. For the parameters without direct measurement (such as *k_plxon_*, *k_plxoff_*), we varied the parameters over a larger range. The parameter ranges are listed below:

| Parameter | Nominal value | Parameter range for random parameter simulations |
| --- | --- | --- |
| $k_{synth}$ | 1.5 nM/min | 0-11.5 nM /min |
| $k_{dest}$ | 0.4 | 0-1 |
| *r* | 1/2 in the first cycle and 1/32 in the subsequent cycles | Systematically change from 1/2, 1/4, 1/8, to 1/21 |
| *k_cdk1on_* | 0.05 | 0-0.5 |
| *k_cdk1off_* | 0.025 | 0-0.5 |
| *p* | 5 | fixed |
| $ec50_{cdc25}$ | 30 nM | 15-45 nM |
| $n_{cdc25}$ | 11 | 5-15 |
| $ec50_{wee1}$ | 35 nM | 20-50 |
| $n_{wee1}$ | 3.5 | 1.75-5.25 |
| $k_{plxon}$ | 1.5 | 0-10 |
| $k_{plxoff}$ | 0.15 | 0-1 |
| $ec50_{plx}$ | 60 nM | 0-100 nM |
| $n_{plx}$ | 5 | Systematically changed from 2, 3, to 6 |
| $k_{apcon}$ | 1.5 | 0-10 |
| $k_{apcoff}$ | 0.125 | 0-1 |
| $ec50_{apc}$ | 0.5 | 0-1 |
| $n_{apc}$ | 4 | Systematically changed from 2, 3, to 6 |

For each chosen parameter, we simulated the temporal evolution of the model for 800 min. We took the simulated traces from the 750 - 800 min, and calculated the standard deviation (SD) and average (avg) of all the data points within this last 50 min window. If the system reached steady state, then we would expect the ratio of AD/avg to be close to 0. We set a cut-off value of 0.1. If SD/avg>0.1, then we saved the parameter as a parameter that generated oscillation. Note that by choosing SD/avg > 0.1, we were ignoring stable oscillations with small amplitudes. However, we believed a small amplitude oscillation is close to no-oscillation in the real biological environment. Therefore, a cutoff of SD/avg>0.1 seemed reasonable.
